# Structural and Proteomic Analysis of the Mouse Cathepsin B-DARPin 4m3 Complex Reveals Species-Specific Binding Determinants

**DOI:** 10.3390/ijms262411910

**Published:** 2025-12-10

**Authors:** Miki Zarić, Livija Tušar, Lovro Kramer, Olga Vasiljeva, Matej Novak, Francis Impens, Aleksandra Usenik, Kris Gevaert, Dušan Turk, Boris Turk

**Affiliations:** 1 Molecular and Structural Biology, Department of Biochemistry, Jožef Stefan Institute, SI-1000 Ljubljana, Slovenia; 2 Jožef Stefan International Postgraduate School, SI-1000 Ljubljana, Slovenia; 3 Centre of Excellence for Integrating Approaches in Chemistry and Biology of Proteins CIPKeBiP, SI-1000 Ljubljana, Slovenia; 4 CytomX Therapeutics, Inc., South San Francisco, CA 94080, USA; 5 VIB-UGent Center for Medical Biotechnology, UGent Department of Biomolecular Medicine, 9052 Ghent, Belgium; 6 Faculty of Chemistry and Chemical Engineering, University of Ljubljana, SI-1000 Ljubljana, Slovenia

**Keywords:** DARPin, cathepsin, protein inhibitor, crystal structure, proteomics

## Abstract

Cathepsin B (CatB) is a lysosomal cysteine protease that plays a major role in various pathologies and is therefore considered a valuable therapeutic target. To address species-specific inhibitor challenges, we characterized the selective binding of designed ankyrin repeat protein (DARPin) 4m3 toward mouse cathepsin B (mCatB) over human CatB (hCatB). The mCatB–DARPin 4m3 complex was validated by size-exclusion chromatography (SEC), nano-differential scanning fluorimetry (nano-DSF), and surface plasmon resonance (SPR), revealing high affinity binding (K_D_ = 65.7 nM) and potent inhibition (Ki = 26.7 nM; mixed competitive/noncompetitive). DARPin 4m3 showed no binding/inhibition toward hCatB. The 1.67 Å crystal structure of the complex—the first for mCatB—identified key interaction residues (e.g., I65/Q66 in mCatB vs. S65/M66 in hCatB) conferring selectivity. Proteomic analysis of endogenous substrates using a support vector machine (SVM) revealed greater similarity between mCatB and hCatB cleavages (Area Under the Curve (AUC) = 0.733) than between mCatB and other human cathepsins (AUC = 0.939–0.965). Clustering and SVM methods offer broadly applicable tools for protease specificity profiling in drug discovery. This study demonstrates the utility of DARPins for species-selective targeting and highlights the importance of integrated structural and proteomic approaches for dissecting protein–protein interactions.

## 1. Introduction

Cathepsin B (CatB), a lysosomal papain-like cysteine protease, regulates protein turnover and antigen processing. In pathological conditions, such as cancer, arthritis, lung disorders, cardiovascular diseases, pancreatitis and hepatitis, CatB is secreted into the extracellular milieu, where it drives disease progression. Critically, CatB inhibition—through genetic ablation or small-molecule inhibitors—significantly impairs disease progression and severity, establishing it as a high-value therapeutic target, including in cancer and acute pancreatitis [1,2,3,4]. Moreover, an inhibitor against cathepsin B was evaluated in Phase I clinical trials for chronic hepatitis [2], although no results were disclosed.

**Figure A1:**
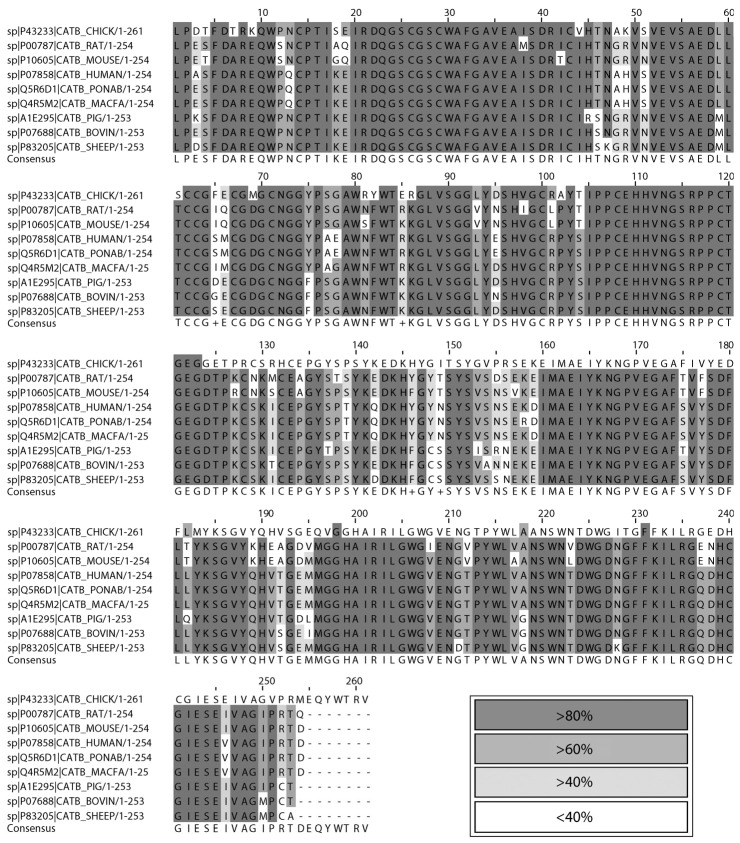
Alignment of amino acid sequences of mature CatB from chicken, rat, mouse, human, orangutan, macaque, pig, bovine, and sheep.

Importantly, species differences may complicate inhibitor development. For example, human cathepsins K [5,6,7,8] and S inhibitors [9,10] show reduced efficacy in rodents due to structural divergence, necessitating the use of costly primate [6,8,11,12] or rabbit models [13,14,15]. Considering these challenges, we investigated whether mouse cathepsin B exhibits similar species-specific divergence that could limit its utility for inhibitor testing. In their mature active forms, human and mouse CatB (hCatB and mCatB) share 83% amino acid identity (Figure A1) and similar biochemical properties, including the K_i_ value for binding to chicken cystatin and the k_on_ value for binding the irreversible CatB inhibitor CA-074 [16]. Despite these similarities, high-resolution structural data and proteomic comparisons are not available, hampering rational inhibitor design.

Designed ankyrin repeat proteins (DARPins) are antibody mimetics exhibiting extreme selectivity and initially served as cocrystallization chaperones [17]. Using a second generation DARPin library, our lab previously developed DARPins 8h6 and 81 through eight rounds of selection against both mouse and human CatB [18]. These cross-species ligands inhibited both orthologs and enabled successful use in in vivo tumor imaging in a mouse breast cancer model [19]. Building on this work, we have developed DARPin 4m3 to achieve mCatB-specific binding, hypothesizing it would reveal subtle interfacial differences undetectable with cross-reactive tools. Here, we resolve the crystal structure of DARPin 4m3 and mouse cathepsin B complex at 1.67 Å, identify selectivity determinants and compare substrate specificities using proteomics data.

## 2. Results

### 2.1. DARPin 4m3 Selectively Binds Mouse and Not Human Cathepsin B

**Figure 1:**
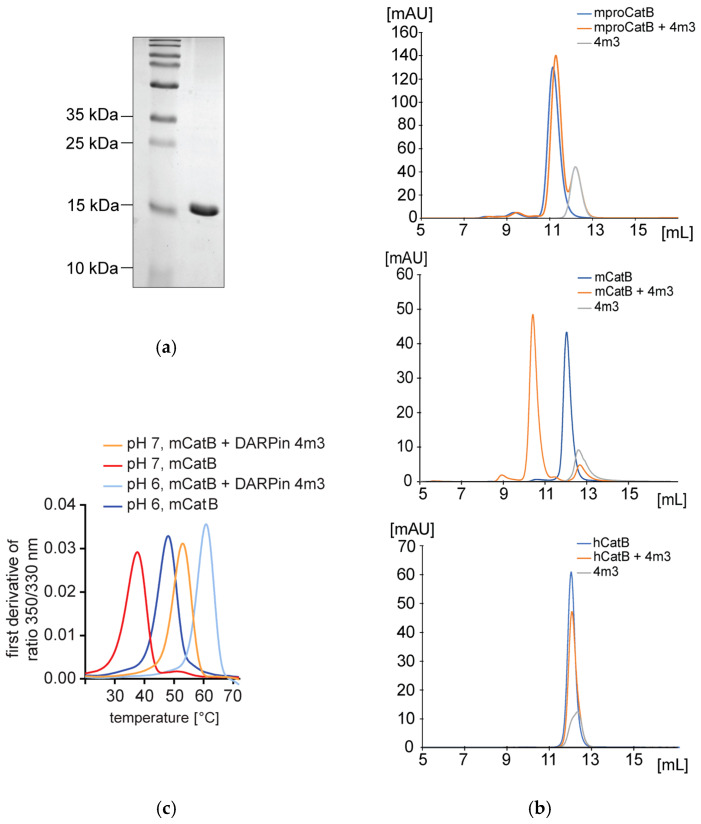
Interaction of purified DARPin 4m3 with mCatB and hCatB. (**a**) SDS-PAGE of the purified DARPin 4m3. (**b**) Analytical size-exclusion chromatography to assess complex formation between DARPin 4m3 and CatB, where the molar ratio of DARPin 4m3 to CatB is 2:1. The blue line represents CatB (mproCatB, mCatB or hCatB). The orange line represents the sample in which DARPin 4m3 and CatB were mixed and incubated. The gray line represents DARPin 4m3 on SEC. (**c**) Nano-differential scan fluorometry analysis of mCatB alone and in complex with DARPin 4m3 at pH 6 or pH 7. The presence of 4m3 thermostabilizes mCatB.

DARPin 4m3 was identified using ELISA screening of clones isolated after four successive rounds of ribosome display selection from the same previously used DARPin library against mouse recombinant CatB [18,19,20] (Figure 1a). Size-exclusion chromatography revealed complex formation between DARPin 4m3 and active mCatB, with an elution peak around 44 kDa (Figure 1b), but no complex formation with hCatB or the inactive mCatB zymogen (mproCatB; Figure 1b). Nano-DSF analysis corroborated binding, showing increased thermal stability of mCatB in the presence of DARPin 4m3 (Figure 1c). At pH 7, mCatB alone aggregated at 37 °C (T_agg_), increasing to 53 °C with DARPin 4m3. At pH 6, T_agg_ increased from 48 °C (mCatB alone) to over 60 °C (complex). Notably, mCatB exhibited greater intrinsic stability at pH 6 than pH 7 regardless of complex formation.

### 2.2. Binding Affinity and Mode of Inhibition of mCatB by DARPin 4m3

**Figure 2:**
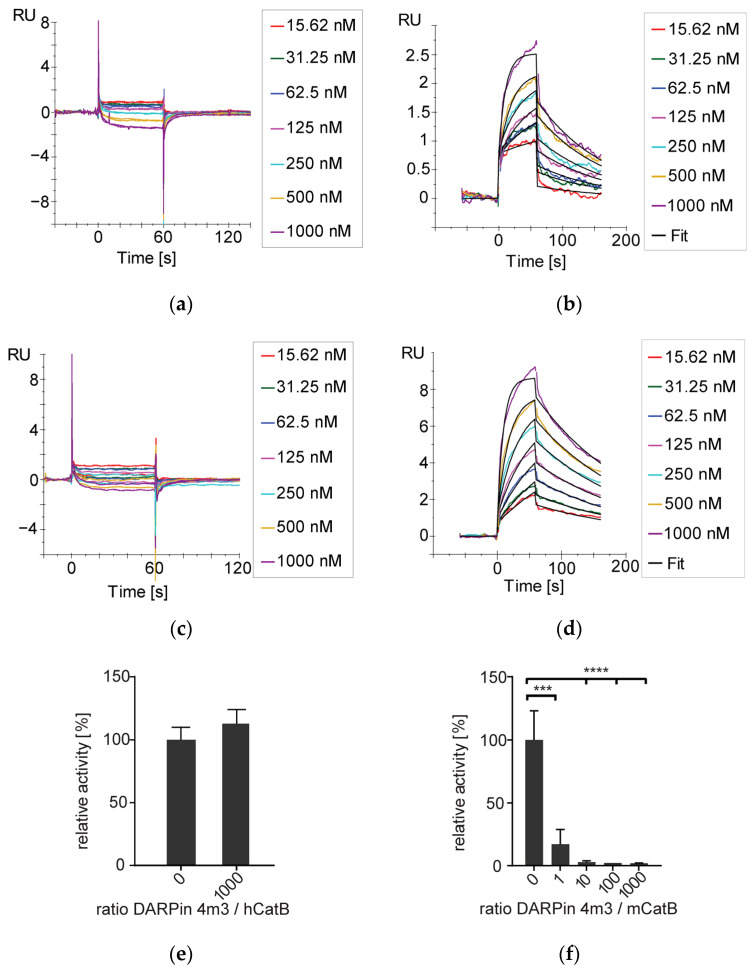
Analysis of DARPin 4m3 binding and inhibition of mCatB and hCatB. (**a**) Surface plasmon resonance analysis of DARPin 4m3 binding to immobilized hCatB at pH 7. (**b**) DARPin 4m3 binding to immobilized mCatB at pH 7. (**c**) DARPin 4m3 binding to immobilized hCatB at pH 6. (**d**) DARPin 4m3 binding to immobilized mCatB at pH 6 (DARPin 4m3 concentration used is shown for panels (**a**–**d**). (**e**) Inhibition assay of DARPin 4m3 against hCatB. Even at a 1000-fold excess, 4m3 did not inhibit mCatB activity. (**f**) Inhibition assay of DARPin 4m3 against mCatB. Reactions were performed at various molar ratios of 4m3 to mCatB. *** (*p* < 0.001) and **** (*p* ≤ 0.0001) denote significant differences between groups according to one-way ANOVA/Tukey’s multiple comparisons tests.

Surface plasmon resonance (SPR) analysis was used to determine the binding affinity of DARPin 4m3 for mCatB. DARPin 4m3 bound to immobilized mCatB at both pH 6 and pH 7, however, showed no interaction with immobilized hCatB (Figure 2). Affinity was slightly higher at pH 6 than pH 7 (K_D_ = 65.7 nM vs. 108.3 nM) (Table 1; Figure 2), in agreement with enhanced complex thermostability at pH 6 observed by nano-DSF (Figure 1c).

**Figure 3:**
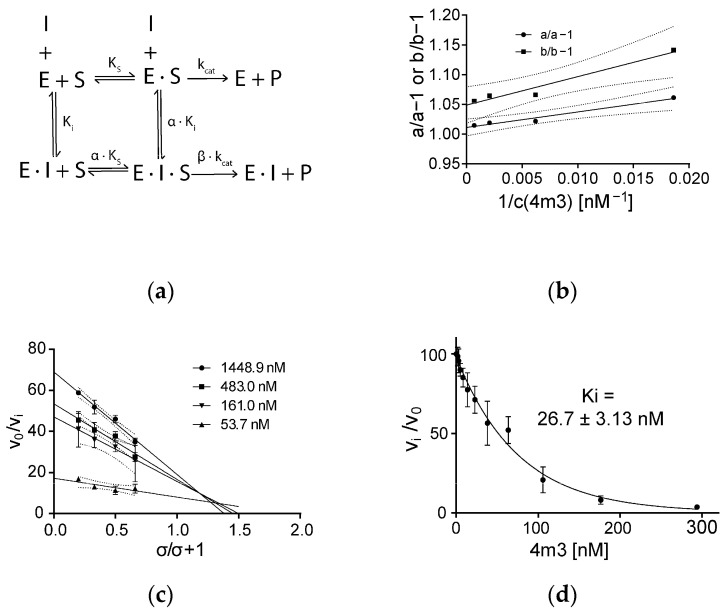
Kinetics of inhibition of mCatB with DARPin 4m3. (**a**) Enzyme inhibition (modulation) scheme according to the general modifier mechanism. (**b**) Primary specific velocity plot for mCatB in the presence of inhibitory DARPin 4m3. (**c**) Secondary specific plot for mCatB in the presence of inhibitory DARPin 4m3. (**d**) Inhibition assay of mCatB (1 nM) by DARPin 4m3 (1.80–294 nM) for determination of K_i_ values. The solid line represents a nonlinear fit using an equation from Szedlacsek et al. [22]. A K_i_ value of 26.7 nM was determined for the interaction.

Activity measurements demonstrated that DARPin 4m3 inhibits mCatB, but not hCatB, even at 1000-fold molar excess (Figure 2e,f), aligning with size-exclusion chromatography data. To elucidate the inhibition mechanism, we used the general modifier scheme (Figure 3a–c) [21] and determined the inhibition constant using the equation for hyperbolic mixed-type inhibition (Figure 3d) [22]:

$$v_{i}=\frac{v_{0}}{2}\cdot \left[1-\frac{\beta \left(1+\sigma \right)}{\alpha +\sigma }\right]\cdot \left\{\begin{array}{c} \sqrt{\left[{\left(\frac{1+\sigma }{\alpha +\sigma }\cdot \frac{\alpha K_{i}}{{\left[E\right]}_{t}}+\frac{{\left[I\right]}_{t}}{{\left[E\right]}_{t}}-1\right)}^{2}+4\cdot \frac{1+\sigma }{\alpha +\sigma }\cdot \frac{\alpha K_{i}}{{\left[E\right]}_{t}}\right]}\\ \frac{+\alpha +\sigma +\beta \left(1+\sigma \right)}{\alpha +\sigma -\beta \left(1+\sigma \right)}-\frac{1+\sigma }{\alpha +\sigma }\cdot \frac{\alpha K_{i}}{{\left[E\right]}_{t}}-\frac{{\left[I\right]}_{t}}{{\left[E\right]}_{t}} \end{array}\right\}$$

Kinetic analysis revealed hyperbolic mixed-type inhibition (α = 1.77 ± 0.042; and β = 0.047 ± 0.0066; Figure 3c). The parameter α, representing the ratio between competitive to noncompetitive inhibition constants, was greater than 1, indicating predominantly competitive inhibition. This confirms competition between DARPin 4m3 and the substrate z-Arg-Arg-AMC for the binding site. We calculated a competitive K_i_ of 26.7 ± 3.13 nM (Figure 3d) and, using α, a noncompetitive K_i_ of 47.25 nM. The parameter β represents the ratio of the turnover rate of the inhibited enzyme to that of the uninhibited enzyme. A β value greater than 0 indicates residual catalytic activity in the enzyme–inhibitor complex, albeit at reduced turnover rates.

**Figure 4:**
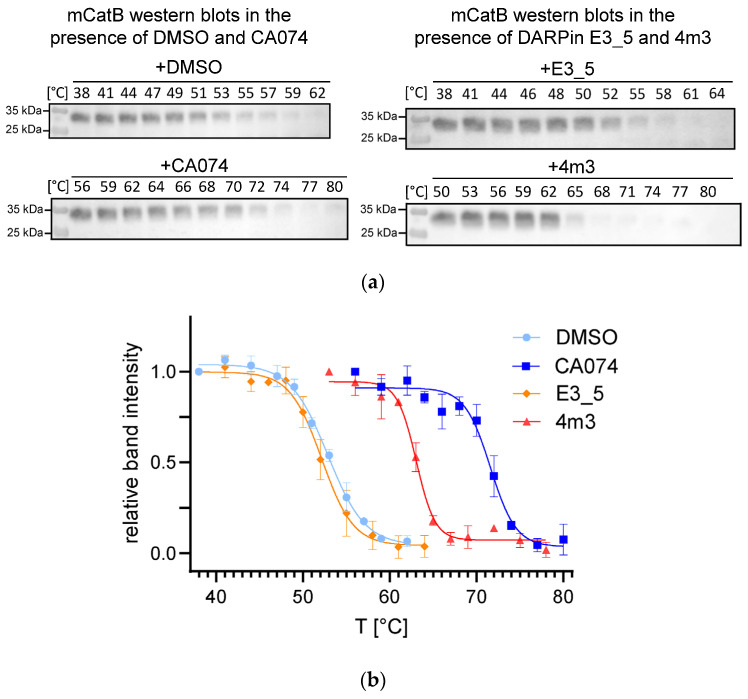
CETSA melting curves (fitted with Boltzmann sigmoid function) for mCatB in RAW 264.7 cell lysates in the presence of various analytes (DMSO, CA074, DARPin E3_5, and DARPin 4m3). Western blots of mCatB, from which band intensities were quantified, are shown above the melt curves. (**a**) On the left, there are examples of Western blots of mCatB after incubation with the selective CatB inhibitor CA074 (+) and its solvent DMSO (−) and on the right, of Western blots of mCatB in the presence of the generic DARPin E3_5 (−) and the mCatB-selective DARPin 4m3. (**b**) Melting curves of mCatB in the presence of the mentioned molecules. Solid lines represent Boltzmann sigmoid fits of the relative mCatB band intensities from the Western blots, which were quantified by densitometry. The apparent T_agg_ of mCatB in the presence of the analytes were 52.2 ± 0.6 °C (DARPin E3_5), 62.9 ± 0.5 °C (DARPin 4m3), 52.9 ± 0.6 °C (DMSO), and 71.6 ± 0.9 °C (CA074).

Further, we performed cellular thermal shift analysis (CETSA) in RAW 264.7 cell llysates, a mouse macrophage cell line, which is known to express high levels of CatB to evaluate DARPin 4m3 binding to endogenous mCatB. DARPin 4m3 successfully stabilized mCatB, increasing its apparent aggregation temperature (T_agg_) from 52.1 ± 0.6 °C (negative control: DARPin E3_5, specific for maltose-binding protein [23] to 62.9 ± 0.5 °C (Figure 4b, Table 2). Western blot quantification using ImageJ [24] confirmed stabilization comparable to the positive control CA-074 (an irreversible CatB-selective E-64 analog; T_agg_ = 71.6 ± 0.9 °C), while DMSO (52.9 ± 0.6 °C; solvent control) and DARPin E3_5 showed no such effect (Figure 4a, Table 2).

### 2.3. Crystal Structure of the Mouse Cathepsin B–DARPin 4m3 Complex

**Figure 5:**
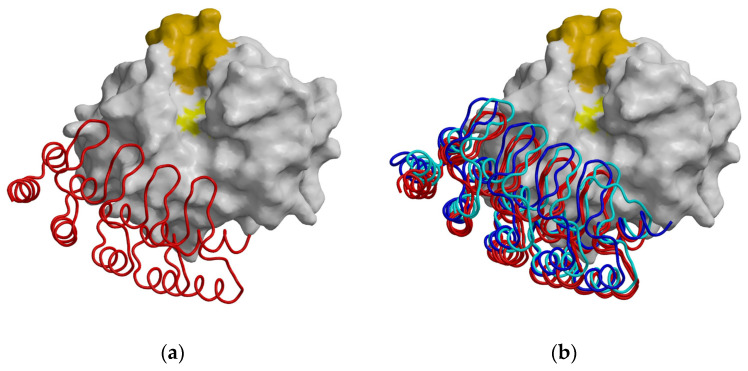
Structure comparison of DARPin binding to cathepsin B. (**a**) Crystal structure of mouse cathepsin B in complex with DARPin 4m3 viewed from the top of the active-site cleft. mCatB is shown as a white surface, with the catalytic residue colored yellow and the occluding loop residues at the top in orange. DARPin 4m3 is shown as a red ribbon. (**b**) Crystal structure of mouse cathepsin B and DARPin 4m3 complex (molecules B and B2, shown in red) superimposed DARPin 81 (cyan) and DARPin 8h6 (blue).

To elucidate differences in DARPins binding between human and mouse cathepsin B, we determined the crystal structure of mCatB in complex with DARPin 4m3 at 1.67 Å resolution (Figure 5a). The complex crystallized in the orthorhombic space group P2_1_2_1_2_1_ with two complexes per asymmetric unit. The structures include residues L1–D254 of mCatB and D2–A160 of DARPin 4m3. Superimposition of Cα atoms yielded RMSDs of 0.37 Å (mCatB monomers), 0.39 Å (DARPin 4m3 molecules), and 0.65 Å (full complexes), indicating only minor crystal-packing effects. For comparison, hCatB monomers (PDB: 1HUC) superimpose with 0.41 Å. Pairwise RMSDs between mCatB and hCatB (0.57–0.64 Å; 83.0% sequence identity) reveal subtle structural differences. Table A1 shows the diffraction data and refinement statistics. Table A2 presents a summary of the results from pairwise structural alignment of complexes between cathepsins B and DARPins.

### 2.4. Structural Comparison of the Cathepsins B, DARPins and Their Complexes

To visualize binding similarities and differences, we superimposed all available DARPin–CatB complex structures (PDB: 5MBM; 5MBL) onto the first pair of molecules in the mCatB–DARPin 4m3 complex. The DARPin molecules revealed overall similar positioning with solvent-exposed catalytic sites, though subtle differences were evident (Figure 5b). Specifically, the mCatB–DARPin 4m3 and hCatB–DARPin 8h6 complexes shared analogous orientations at the interdomain interface of the enzyme and L-domain surface, maintaining exposure of the Cys–His catalytic dyad (Figure 5b).

**Figure 6:**
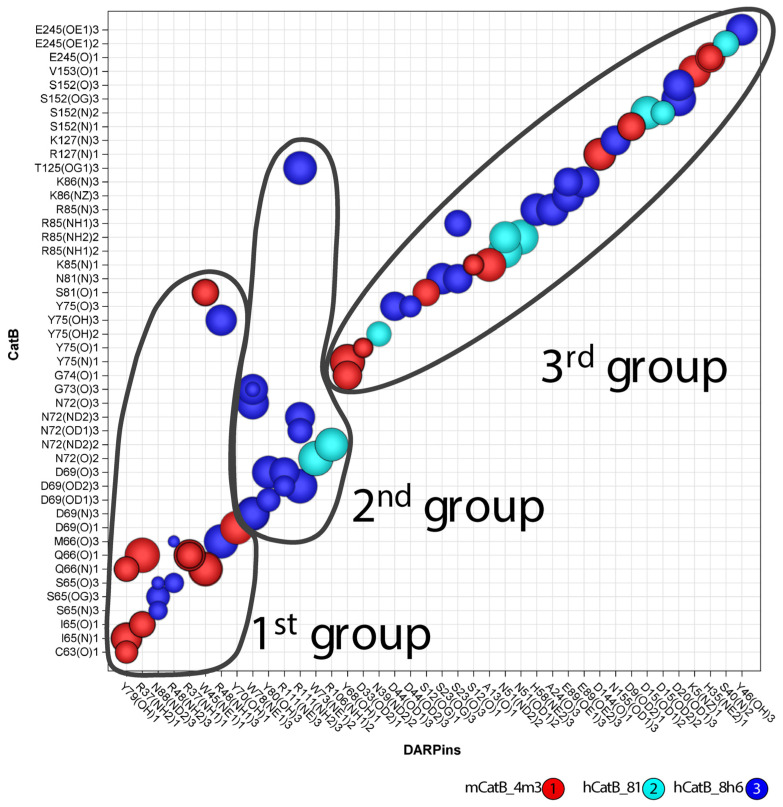
Interacting residues of mCatB/hCatB and DARPins. Each circle denotes interactions between cathepsin and DARPin residues on the vertical and horizontal axes. Circle colors (red, cyan, and blue) correspond to the mouse and human complexes, as indicated in the bottom right corner. The circle radius represents interaction distance: smaller circles indicate closer interactions, while larger circles indicate longer interactions.

To map interface differences, we created a 2D interaction table comparing DARPin binding across species. While 11 residues in mCatB and hCatB interacted with 11 residues in DARPin 4m3 and 8h6, only 6 hCatB residues interacted with 6 residues in DARPin 81. Color coding revealed three interaction groups (Figure 6). Group 1 (shared between mCatB/hCatB and 4m3/8h6) featured key mCatB residues C63, I65, and Q66 binding DARPin 4m3 Y79, R37, and W45, respectively, while in hCatB, S65 and M66 interacted with DARPin 8h6 N88 and R48. Position 66 thus showed additional divergence (mCatB Q66 vs. hCatB M66). Group 2 (exclusive to hCatB with 8h6/81) involved hCatB N72 interacting with DARPin 8h6 R111 and DARPin 81 W73, R106, plus hCatB G73 and T125 binding DARPin 8h6 W78 and R111. Group 3 contained nearly identical residues conserved across all complexes.

**Figure 7:**
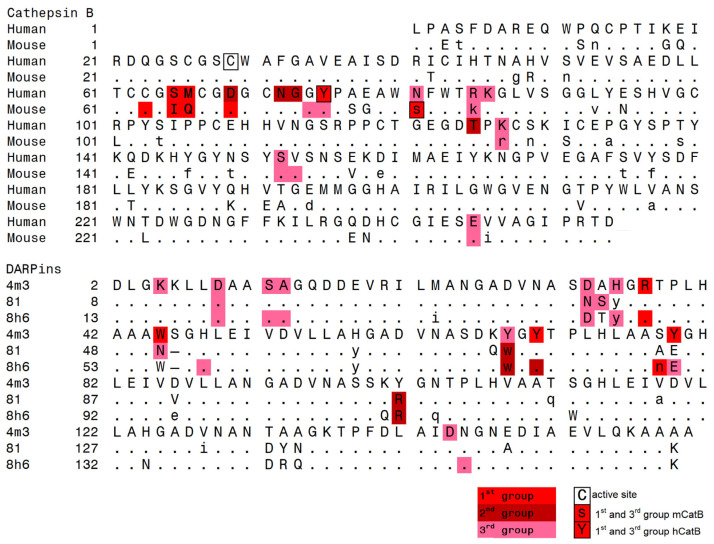
Structure-based alignment of mCatB, hCatB, and DARPins generated with FatCat [25] and MAIN [26]. Identical residues are written as dots, homologous residues as lowercase letters, and different residues as uppercase letters. The active site C29 and hCatB Y65, and mCatB S81 are highlighted with frames. Residues forming interactions between cathepsin and DARPin are colored red, dark red, and Indian red, corresponding to the three interacting groups defined in Figure 6. Residues labeled Y and s indicate interactions that split between two groups.

To correlate interaction interfaces with sequence divergence, we mapped the interacting residues onto a sequence alignment of mouse and human CatB (Figure 7), color-coding them by interaction group (shades of red). The primary contact region (residues S66 to K87 in hCatB and C63 to K86 in mCatB) coincided with the area of greatest sequence divergence. Only minor sequence differences occurred outside this interface. Consistent with the interaction differences analysis (Figure 6), the most significant variations occurred at positions 65 and 66: Ile and Gln in mCatB versus Ser and Met in hCatB. Notably, hCatB N72 formed contacts with DARPin 8h6 R111 and DARPin 81 residues W73 and R106. These interactions were absent in mCatB, where tyrosine (Y101) in DARPin 4m3, a smaller residue compared to R111 in DARPin 8h6 or R106 in DARPin 81, occupies the structurally equivalent position (Figure 7).

To assess the impact of the I65/Q66→S65/M66 substitution on the mCatB-DARPin 4m3 interface, we performed protein–protein docking simulations using ClusPro 2.0 [27]. Both wild-type and mutant complexes were docked under identical conditions. Analysis of the top-ranked docked complexes revealed minimal changes in interfacial hydrogen bond networks upon mutation (Table A6 and Table A7). However, we observed slight differences in ClusPro docking scores, particularly when the interaction region was not specified (average difference 0.5, standard deviation 4.6; Table A5, IDs 1358636 & 1358637), with larger differences noted when the interaction region was specified (average difference 1235.8, standard deviation 52.8; Table A5).

Interaction energies, including van der Waals and electrostatic interactions, were also calculated for our complex and for the four top-ranked ClusPro docking models of both wild-type and mutated mCatB (Table A8) using MAIN [26]. Our complex showed interaction energies of −425.3554 (molecules A/B) and −538.8692 (molecules A2/B2). For the mCatB wild-type complex, the lowest total energy (−344.8188) was obtained with the 006_00 docking model (docking ID 1357926), representing a difference of −80.5366 and −194.0504 compared to the A/B and A2/B2 models, respectively. The lowest energy (−748.2675 energy unit) was obtained with the 000_00 docking model. For the mutated mCatB complex, the lowest energy (−416.8979) was obtained with the 000_00 docking model (docking ID 1357923), while the 002_00 docking model yielded the highest energy (−548.9533).

**Figure A2:**
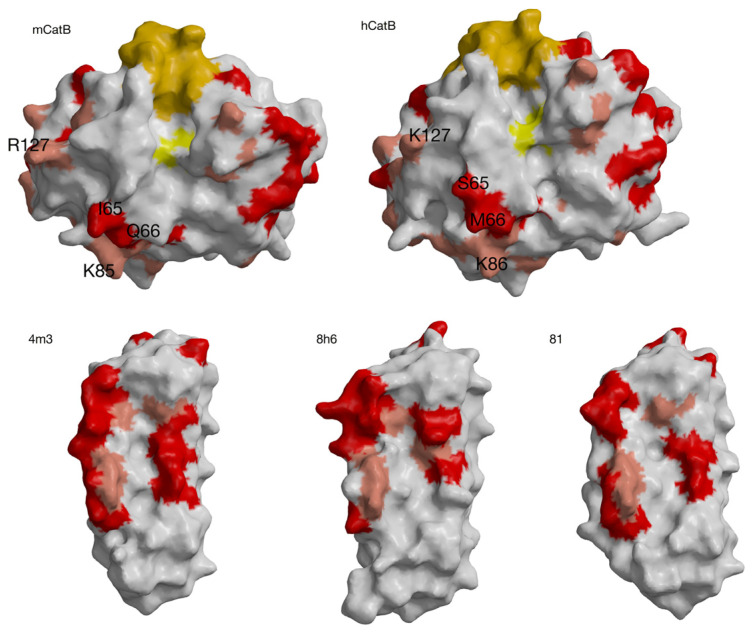
Structure-based alignments of mCatB and hCatB and DARPins with MAIN [26]. Areas with different amino acids are shown in red and homologs are shown in tomato red. The active site is colored yellow and the amino acid residues of the occlusion loop from 108 to 119 are colored orange. Residues involved in the interaction between cathepsin B and DARPins are indicated by a one-letter code and a number. Protein structures were generated using MAIN software [26] and rendered using Raster3D [28].

**Figure A3:**
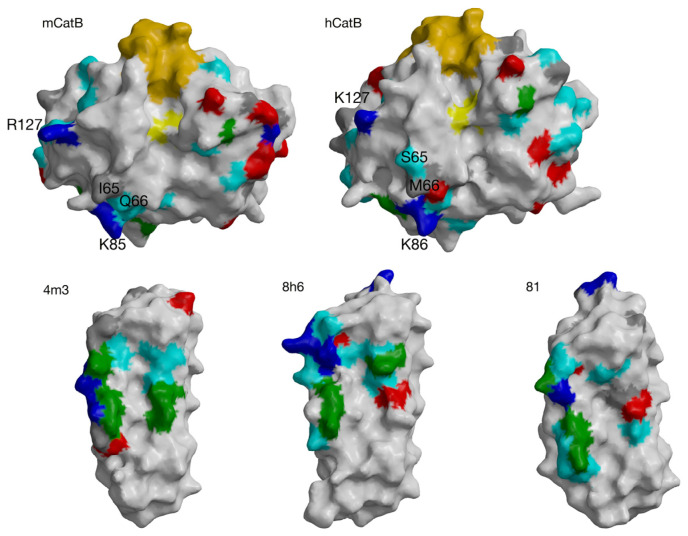
Structure-based alignments of mCatB and hCatB and DARPins with MAIN [26]. The colors of the different residues and homologs represent their chemical properties, namely the hydrophobic A, G, I, V, L, P, M, and C (dark gray), the hydrophilic S, T, N, Q (cyan), the negatively charged D, E (red), the positively charged K, R (blue) and the aromatic hydrophobic Y, F, W, H (green). The CSO29C is colored yellow, and the amino acid residues of the occlusion loop from C108 to C119 are colored orange. Residues involved in the interaction between cathepsin B and DARPins are indicated by a one-letter code and a number. The protein structures were generated with MAIN [26] and rendered using Raster3D [28].

These analyses suggest the I65/Q66→S65/M66 substitution induces subtle changes in the energetic landscape of the DARPin interaction (Figure A2 and Figure A3). The substitution of hydrophobic/polar residues to polar/hydrophobic residues may contribute to local structural divergence near the active site but does not appear to be a primary determinant of DARPin binding energetics. While docking simulations [27] and MAIN calculations [26] provide estimates of binding affinity and are not equivalent to experimental determination of interaction constants, these in silico analyses support the hypothesis that the I65/Q66/S65/M66 residues contribute to the energetic stability of the DARPin interaction.

**Figure 8:**
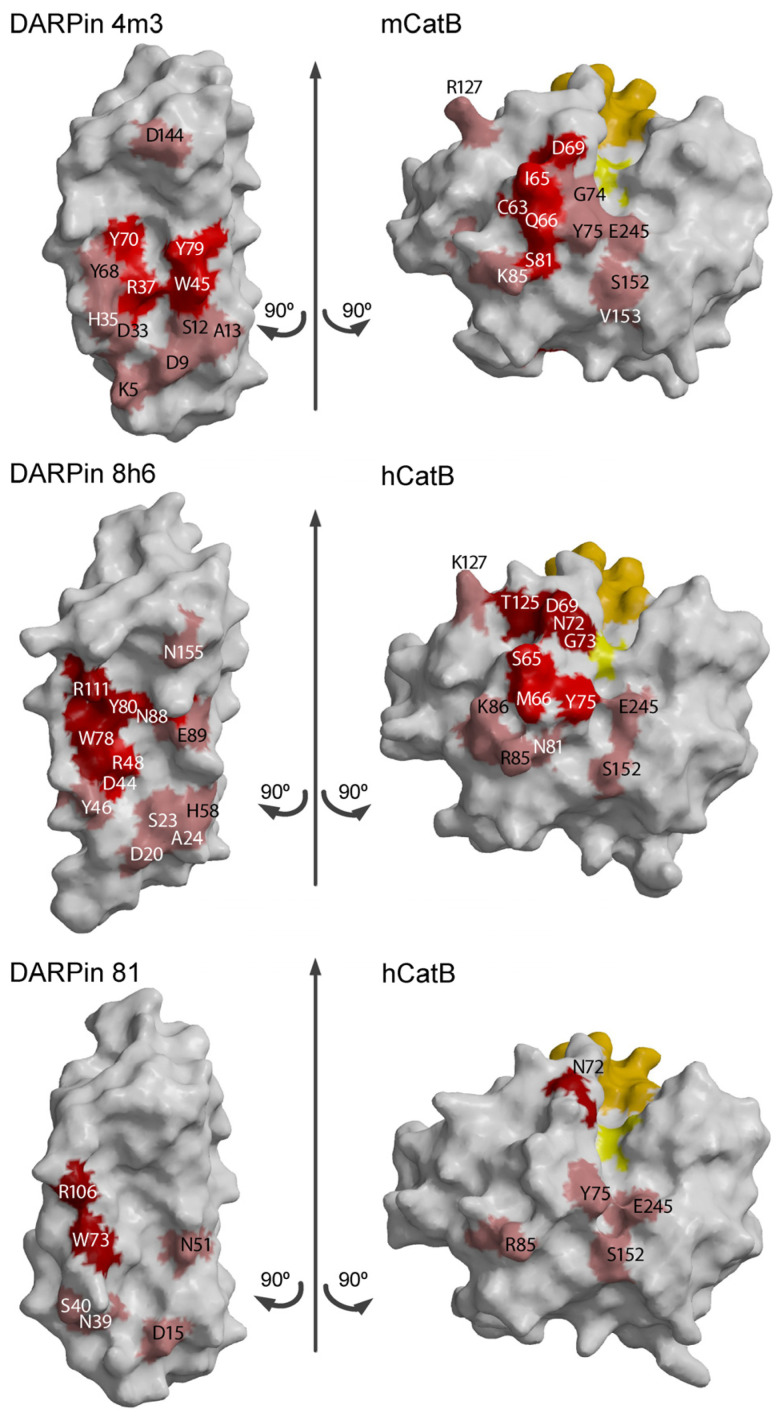
3D visualization of the interacting residues between mCatB/hCatB and DARPins, divided into three groups (red, dark red, and Indian red) as defined in Figure 7. The active site residue is shown in yellow, and the occluding loop residues are shown in orange. Protein structures were generated with MAIN [26] and rendered with Raster3D [28]. Arrows indicate the direction of the rotation of the complex subunits.

Rotating the structures of the complex by ±90° with minor spatial adjustments visualizes the interaction groups by mapping their color codes from Figure 7 onto the molecular surfaces (Figure 8).

### 2.5. Substrates as Indicators of Differences Between Mouse and Human CatB

To compare the specificity between mouse and human CatB, we performed mass spectrometry analysis of MEF cell lysates treated with mCatB using the same approach as before. The data for human cathepsins K, V, B, L, S, and F were also taken from the same study [29].

**Figure 9:**
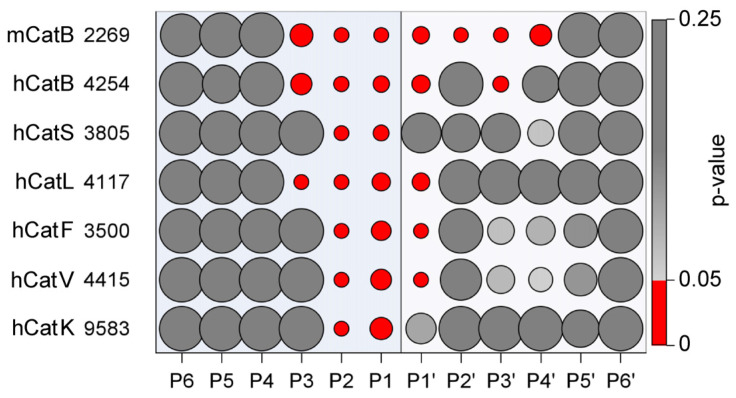
Normality of substrate residue distributions. The *p*-values for the normality of the distributions of the residues at the positions from P6 to P6′ (columns) for each cathepsin (rows) are indicated by the size of the circles. The red, light gray, and gray circles indicate *p*-values ≤ 0.05 (not normal), >0.05 and ≤0.08 (normal but close to the limit 0.05), and >0.08, respectively.

The peptides/substrates data were then analyzed using Schechter-Berger nomenclature. The Anderson–Darling test revealed non-normal residue distributions (heterogeneous positions) at 7 substrate positions (P3–P4′) for mCatB (Figure 9; visualized structurally in Figure A5), compared to 5 positions for hCatB. This suggests mCatB engages more substrate-binding subsites during catalysis.

**Figure 10:**
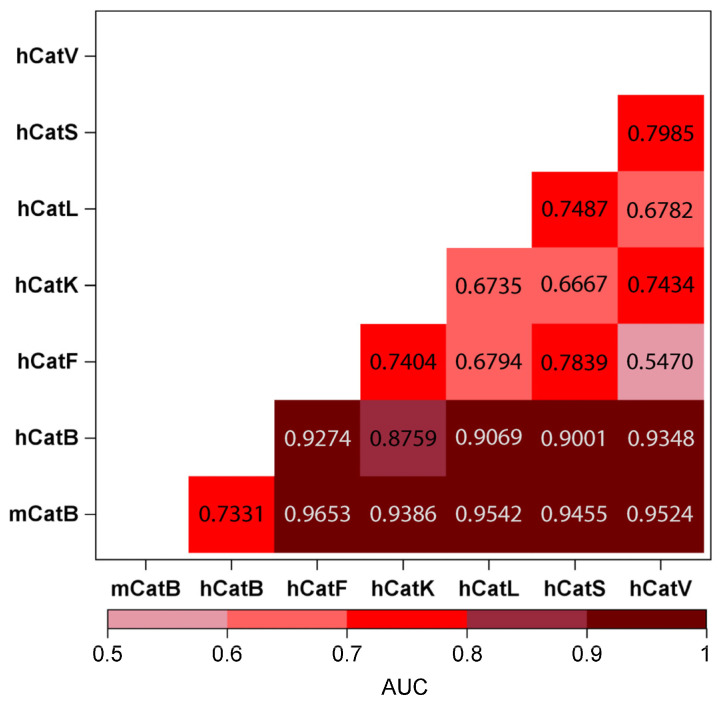
Calculated AUC values for pairwise comparisons of cleavage sites from different cathepsins for the P3–P4′ region, which proved most informative for distinguishing cathepsin cleavage site specificities. Values between 0.5 and 0.6 (light pink) indicate the most similar substrate groups; 0.6 to 0.7 (rose), 0.7 to 0.8 (red), 0.8 to 0.9 (deep pink), and 0.9 to 1.0 (maroon) represent progressively greater differences, with maroon indicating the most distinct groups.

**Figure A4:**
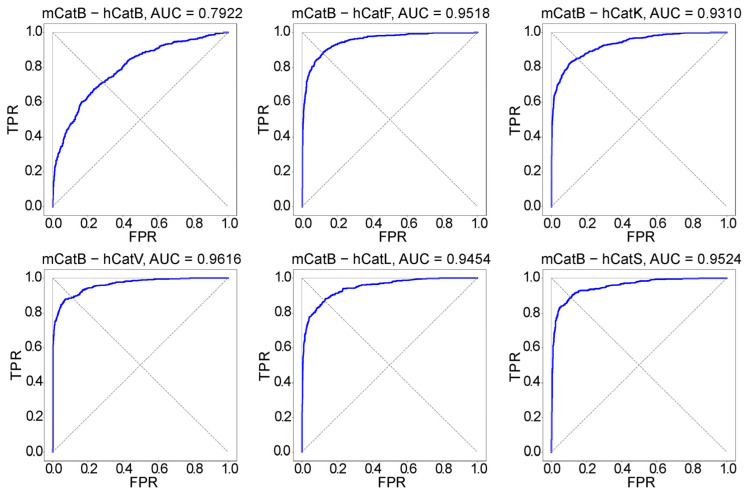
ROC curves and calculated AUC values for the positions from P4 to P4′ for combinations of substrates of mouse cathepsin B (mCatB) with separate substrates of human cathepsin B (hCatB), human cathepsin F (hCatF), human cathepsin K (hCatK), human cathepsin L (hCatL), human cathepsin V (hCatV), and human cathepsin S (hCatS) (see also Table A3).

**Figure A5:**
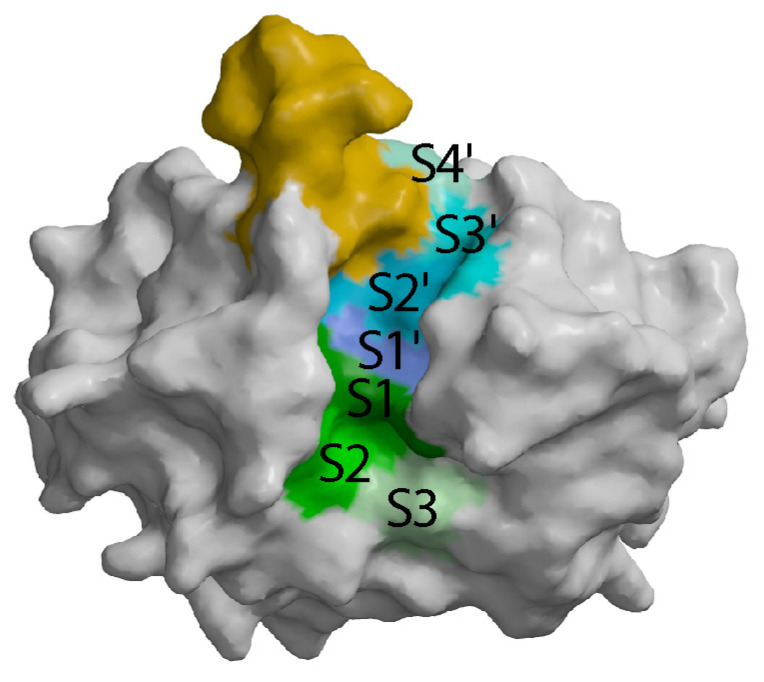
The positions S3–S4′ for the binding of substrates are shown in mCatB: prime sites S1′–S4′ (blue shading) and non-prime sites S1–S3 (green shading). Residues at the sites: S1′ 23, 176, 199; S2′ 24, 33, 221; S3′ 180, 181, 225, 231; S4′: 22, 224; S1 26, 27, 29, 71, 198; S2 30, 73, 74, 200, 245; S3 76, 173, 245. Positions were determined based on inhibitors bound to human cathepsin B.

In our previous study, we used Support Vector Machine learning algorithms to predict cleavage sites by differentiating cleaved and non-cleaved sequences [29]. Modeled performance was quantified using the area under the Receiver Operating Characteristic curve (AUC-ROC). Here, we repurpose AUC as a similarity metric for pairwise comparisons of cathepsin cleavage datasets for a different goal: Discrimination of two datasets. The AUC values for pairwise comparisons of substrate cleavage data sets across cathepsins were thus calculated by selecting different ranges of positions: P3–P4′ (Figure 10, Table A3), P4–P4′ (Figure A4, Table A3), and P15–P15′ (Table A3). The differences between the peptides of mCatB vs. hCatB and human cathepsins K, V, L, S, and F are most obvious when positions P3–P4′ and P4–P4′ are considered (Table A3). The lower AUC values indicate greater similarity between two data sets (reduced discriminative power), while higher AUC values reflect greater dissimilarity (enhanced discriminative power). The color gradient (light pink towards maroon red shades) represents increasing dissimilarity (higher AUC). Mouse and human CatB datasets showed strong similarity (AUC = 0.73), yet they remained distinguishable from each other and formed a distinct cluster relative to the datasets of other cathepsins (Figure 10). Notably, hCatV and hCatF data sets exhibited highest similarity (lowest AUC, light pink).

The discrimination of mCatB and hCatB cleavage sites encouraged us to seek substrates specific to hCatB or mCatB. To identify species-specific substrates, we clustered the combined cleavage data sets. Using a maximum of 400 clusters, we obtained 22 clusters containing 227 substrates (Supplementary Data S1). These included 18 clusters (218 substrates) specific to hCatB and 4 clusters (9 substrates) specific to mCatB (Table A4). The hCatB-specific substrates represented 161 unique protein sequences, including actins, tubulins, kinases, ribosomal proteins, histones, and nucleolar proteins (Supplementary Data S1). This further supports the idea that mouse and human cathepsin B do not have entirely identical physiological roles, although the differences in substrate list may in part reflect the different types of cell lines used in the proteomic analysis—mouse embryonic fibroblasts (MEFs) vs. human neuroblastoma cell line (SH-SY5Y).

## 3. Discussion

Cathepsin B has been one of the most studied human cysteine cathepsins, especially after it was found to be linked with cancer and arthritis. In addition to its direct therapeutic potential, the enzyme was widely targeted for prodrug and antibody–drug conjugate activation [30,31,32,33] and evaluated for diagnostic imaging as well as for target for targeted drug delivery in cancer animal models [1,2,19]. However, despite a vast amount of information about human cathepsin B [34,35,36], little is known about mouse cathepsin B [16], including a detailed comparison of cross-species efficacy.

To address this gap, we determined the crystal structure of mCatB bound to the inhibitory designed ankyrin repeat protein (DARPin) 4m3. This highly potent and selective inhibitor exhibits nanomolar affinity for mCatB but no detectable binding to hCatB, demonstrating that species-specific targeting is achievable despite high structural similarity. DARPin 4m3 also had a greater stabilizing effect (Figure 1c) and greater affinity (Figure 2b,d) for mCatB at pH 6 than at pH 7, which agrees with an earlier study that showed large pH-induced structural changes already at pH 7.4 and 37 °C [37]. Structure analysis reveals DARPin 4m3 binds adjacent to but does not occlude the mCatB active site cleft, mirroring the binding mode of DARPins 8h6 and 81 on hCatB. Consistent with this, kinetic studies indicate hyperbolic mixed-type inhibition (Figure 3b,c), where the inhibitor-bound enzyme retains partial catalytic activity toward small substrates like z-RR-AMC that can enter the cleft and be processed. Similar inhibition mechanisms occur with DARPins 8h6 and 81 targeting hCatB and DARPin AR_F8 targeting caspase-2 [19,38].

DARPin 4m3 binding contacts occurred at the regions which were most different between mCatB and hCatB, indicating that the engineering of the selectivity of DARPin 4m3 addressed the most potent region of the differences (Figure 7 and Figure A1). Notably, three distinct interaction groups at the DARPin interface (Figure 6) explain the high selectivity of 4m3, highlighting the potential of DARPin scaffolds to exploit subtle ortholog differences. While active site subsites show identical residues (Figure A2 and Figure A3), substrate specificity analysis via SVM-based machine learning reveals divergent cleavage preferences between mCatB and hCatB, albeit with greater mutual similarity (AUC = 0.73 than CatL or CatV (AUC = 0.82–0.89) (Figure 10). This aligns with their closer phylogenetic relationship [39]. Clustering identified species-specific cleavage patterns (Supplementary Data S1), offering a pathway for developing selective substrates. This approach could extend to other proteases, pending broader proteomic data sets.

Collectively, mCatB and hCatB show sufficient functional divergence to warrant careful preclinical model selection. Our mCatB structure enables rational design of murine-specific probes and inhibitors [40], facilitating translational research. Critically, species-selective inhibitors like DARPin 4m3 can dissect host-derived CatB roles in xenograft tumor models [3,41], distinguishing tumor-cell versus microenvironmental contributions [42,43]. Thus, while mCatB remains valuable for human CatB research, its differences necessitate context-specific validation.

## 4. Materials and Methods

### 4.1. Expression and Purification of Recombinant Proteins

DARPin 4m3 was selected using four rounds of ribosomal display against mouse cathepsin B, as previously described [19]. For expression, the DARPin 4m3 sequence was cloned into the pET22b+ vector. Due to degradation issues during expression, the 6×His-tag was relocated to the C-terminus. The Rosetta gami B(DE3)pLysS strain was transformed using this construct. Cultures grew in Terrific Broth with appropriate antibiotics at 37 °C until OD_600_ reached approximately 1.5. Flasks were then transferred to 4 °C for 15 min, and expression was induced by the addition of 1 mM IPTG. Expression was then continued at 18 °C for 20 h and 300 rpm.

Sample preparation for Ni-NTA chromatography followed established protocols for cathepsin purification [44]. Ni-NTA purifications were followed by size-exclusion chromatography on a Superdex 75 column in 20 mM HEPES, 200 mM NaCl, pH 7.4.

Human and mouse CatB were expressed as soluble proteins in *Escherichia coli* strain Rosetta gami B(DE3)pLysS, as previously described [44]. The enzymes were then active-site titrated using the broad-spectrum cysteine cathepsin inhibitor E64 as described earlier [45].

### 4.2. Inhibition Assay

We measured the activity of human and murine CatB toward the substrate z-RR-AMC in the presence of DARPin 4m3. Enzyme solutions (10 nM each) were mixed with DARPin 4m3 (present at 1- to 1000-molar excess over CatB) and incubated at 37 °C for 15 min in cathepsin activity buffer (100 mM sodium phosphate buffer, 5 mM DTT, 1 mM EDTA, 0.1% PEG6000, pH 6.0) before cathepsin activity was measured in a microplate reader at 37 °C as described before [19].

The inhibition mechanism of DARPin 4m3 against mCatB was determined using the general modifier scheme [21]. The K_m_ of z-RR-AMC for mCatB was determined to be 547.7 µM. The enzyme concentration (mCatB) in the assay was 1 nM, while the substrate and inhibitor concentrations varied. Substrate concentrations (z-RR-AMC) ranged from 135 µM to 1080 µM, whereas inhibitor (DARPin 4m3) concentrations ranged from 53.7 nM to 1448.9 nM.

For inhibition constant (K_i_) determination, 1 nM mCatB was incubated with DARPin 4m3 (1.8 nM to 294 nM) for 15 min at 37 °C in cathepsin activity buffer. Initial reaction rates against 700 µM z-RR-AMC were measured and nonlinearly fitted as described by Szedlacsek et al. [22] for the hyperbolic mode of inhibition. This fit can be used as a standard for any mechanism, even in the absence of tight binding conditions. Fitting and graphical analyses were performed using the GraphPad Prism5 for Windows (GraphPad Software, Boston, MA, USA).

### 4.3. Nano-Differential Fluorimetry Analysis

Protein samples were diluted to 0.5 mg/mL in 100 mM PBS (pH 6 and 7). Measurements were made in the range of 20 to 95 °C with a ramp rate of 1 °C/min by monitoring the intrinsic tryptophan fluorescence absorbances at 350 and 330 nm on Nanotemper Prometheus (NanoTemper Technologies GmbH, München, Germany). The manufacturer’s software calculated the fluorescence intensity ratio and its first derivative (PR.Stability Analysis, v1.1).

### 4.4. Surface Plasmon Resonance

SPR was performed with Biacore T200 (Cytiva, Marlborough, MA, USA) on CM5 sensor chips. Mouse and human CatB (100 nM) was immobilized on the chip at 30 µL/min until it reached 700 response units (RU). After immobilization, seven different concentrations of DARPin 4m3 were then run over the chip. Results were analyzed using the Biacore T200 Kinetics Summary software (v3.2). Assays were performed in sodium phosphate-buffered saline (20 mM Na-phosphate, 150 mM NaCl) at pH 6 or 7 and 25 °C.

### 4.5. Analytical Size-Exclusion Chromatography

Analytical SEC was used to check the interaction between DARPin 4m3 and CatB. Samples were diluted in 20 mM sodium phosphate, 150 mM NaCl, pH 7.2. DARPin 4m3 (20 mM) and CatB were mixed in a 2:1 molar ratio, then incubated at approximately 20–22 °C for 10 min and centrifuged at 20,000× *g* for 10 min. Samples were run at a flow rate of 1 mL/min at room temperature.

### 4.6. Cellular Thermal Shift Assay

The CETSA was performed as previously described [46]. Briefly, RAW 264.7 cells were pelleted and resuspended in cathepsin activity buffer (100 mM sodium phosphate, 1 mM EDTA, 0.1% PEG 6000, 5 mM DTT, pH 6). The cell suspensions were subjected to three freeze–thaw cycles in liquid nitrogen and centrifuged at 32,000 RCF for 20 min at 4 °C. The protein content of the supernatant was measured using the Bradford assay. Aliquots were treated with 10 µM of the specified analyte and incubated at room temperature for 10 min. Samples were then exposed to a specified temperature in a preheated PCR machine for 3 min, followed by incubation at room temperature for 3 min and then snap-frozen. After thawing at room temperature, samples were centrifuged again at 32,000 RCF for 20 min at 4 °C. The supernatant was transferred to new tubes and analyzed by Western blot using anti-mouse CatB antibodies. Band intensities were quantified using ImageJ software, v1.53k [24]. To obtain apparent aggregation temperatures (T_agg_), band intensities were fitted in GraphPad Prism using the Boltzmann sigmoidal equation.

### 4.7. Structure Determination

The complex of DARPin 4m3 and mouse CatB was formed by mixing at a molar ratio of 3:2 in 20 mM MES, 30 mM NaCl, pH 6, and incubating for 15 min at room temperature. The complex was then purified by size-exclusion chromatography on a Superdex 75pg (Cytiva, Marlborough, MA, USA) at 4 °C with a flow rate of 1 mL/min. The sample was concentrated to approximately 24.6 mg/mL using Amicon Ultra-15 Centrifugal Filter Units (Merck, Darmstadt, Germany) with a cut-off of 10 kDa. Crystals were grown using the sitting-drop vapor method in 0.1 M Bis-Tris, 20% PEG3350, 0.2 M sodium acetate, and pH 6.5. The ratio of protein solution to mother liquor was 2:3 (5 µL drops). The crystals grew for one week. They were then soaked in mother liquor containing 10% glycerol and frozen in liquid nitrogen until data collection. Diffraction data were obtained at the Elettra synchrotron, XRD2 beamline, in Trieste, Italy.

The seven data sets with resolutions ranging approximately from 1.7 to 3.6 Å were collected and processed using XDS software (release 2022, built = 20220220) [47]. The complex structure was solved by molecular replacement with Phaser [48], using PDB ID entry 5MBM as a model. The space group was P2_1_2_1_2_1_, and the resolution was 1.67 Å. The data collection and refinement statistics are presented in Table A1. It contains two molecules in the asymmetric unit, referred to as molecules A and B (the calculated RMSDs are presented in Table A1). The MAIN software (release 2025) [26] was used for map calculation, model building, refinement, and validation. For refinement, the maximum likelihood free kick target function (ML) was used, which applies all structural factors instead of part of the test data set to calculate phase error estimates [49]. The CatB–DARPin complex was deposited in the Protein Data Bank (PDB).

### 4.8. Data Sets of Human Cathepsins K, V, L, S, F, and mCatB Substrates

All the cleavage sites for human cathepsins K, V, L, S, and F were taken from our previous study [29]. The procedure was essentially the same as described earlier, except that mouse embryonic fibroblasts (MEF) were used as a source of mouse proteins. Mouse cells were used to ensure better matching with the mouse protease, as it was shown that substrates and proteases have co-evolved during evolution [50]. Lysates of MEFs were treated with mouse cathepsin B and the cleavages were determined by consecutive reverse phase high-performance liquid chromatography (RP-HPLC) and liquid chromatography–tandem mass spectroscopy (LC-MS/MS) on an LTQ Orbitrap XL or Orbitrap Velos mass spectrometer (Thermo) using the N-terminal combined fractional diagonal chromatography (COFRADIC) protocol. All the cleavage sequences were then identified in the same way as described [29]. In total, 2269 cleavage sites were identified, which is less than for human cathepsin B (4254 cleavage sites).

### 4.9. Evaluation of Substrate Differences Between mCatB and Human Cathepsins B, K, V, L, S, and F Using Support Vector Machine Algorithm and Clustering

Data analysis was performed using our SAPS-ESI (Statistical Approach to Peptidyl Substrate–Enzyme-Specific Interactions) software platform, developed with Python 3.8 and SAS for Windows 9.4 [29,51]. A total of 2269 cleavage sites were analyzed for mCatB and 4254 for hCatB, while human cathepsins K, V, L, S, and F had 9583, 4415, 4117, 3805, and 3500 cleavage sites, respectively, for comparative analysis. First, the distribution of amino acid residues near cleavage sites was evaluated using the Anderson–Darling normality test. Positions with non-normal residue distribution or heterogeneous positions are marked with small red circles (*p*-value less than or equal to 0.05) or light gray circles (*p*-values greater than 0.05 and less than or equal to 0.08) or dark gray (*p*-values greater than 0.08) when the distribution of residues at specific positions is visualized (Figure 9).

Previously, we used the SVM algorithm to predict the cleavage sites of cathepsins K, V, B, L, F, S, and V based on cleaved and non-cleaved 8-amino-acid-long peptides. Here, we used the SVM algorithm to classify the two groups of cleavage sites of two different cathepsins based on AUC-ROC, a measure of classification performance. This is a graphical representation that illustrates the performance of a binary classification model at different thresholds. A larger AUC indicates the ROC curve of an excellent binary model (ideal at AUC = 1.0) that can perfectly classify two selected groups. Conversely, a smaller AUC below 75% indicates similar groups of cleavage sites of two cathepsins.

Pairwise similarity analyses were performed in Python 3.8 using scikit-learn with a Support Vector Machine (SVM) classifier. Cleaved sequences of mCatB were compared with those of hCatB, hCatK, hCatV, hCatL, hCatS, and hCatF, as well as between selected human cathepsins. For each pairwise comparison, we defined positive and negative datasets. For example, cleaved sequences of mCatB were labeled as positive (code “1”), and cleaved sequences of hCatB were labelled as negative (code “0”).

We examined three sequence regions around the cleavage site—P3–P4′, P4–P4′, and P15–P15′—to identify which range best differentiates cathepsin pairs. Figure 9 illustrates these positions, where red markers indicate positions with residues contributing most to the differences between cathepsins. Specific positions were determined using the Anderson–Darling normality test, identifying positions with reduced amino acid variability (typically one or two predominant residues [29]).

**Figure A6:**
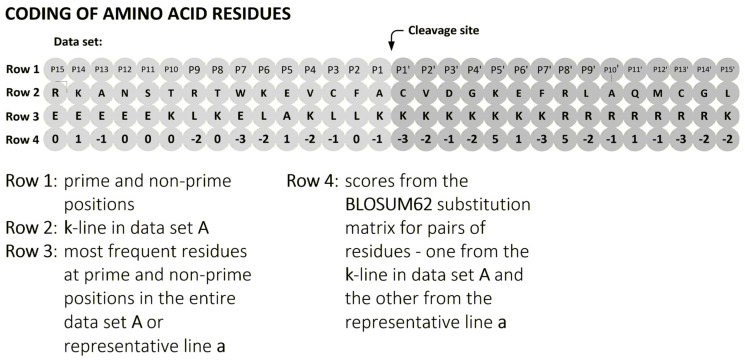
Coding of amino acid residues using the BLOSUM62 substitution matrix.

Selected residues were encoded as numerical features using BLOSUM62 substitution scores (Figure A6). Each residue pair (e.g., A vs. K at position P1) was assigned its corresponding BLOSUM62 value.

Model training and validation were performed using a 25-fold jackknife cross-validation approach. In each iteration, 25% of the cleavage sites were reserved for testing, and the remaining 75% were used for training. This process was repeated multiple times with different random splits to ensure that most data points served in both training and testing across iterations. The maximum number of training iterations was 200,000.

SVM hyperparameters were optimized by grid search, testing the following ranges:

- Regularization parameter C = [0.001, 0.01, 0.1, 1]
- Kernel = [‘linear’, ‘rbf’, ‘poly’, ‘sigmoid’]
- Gamma = [‘scale’, ‘auto’]
- Class weight = [‘balanced’, ‘none’]
- Jackknife fraction = [0.10, 0.25]

The optimal combination—yielding the highest AUC—was: kernel = ‘rbf’, gamma = ‘scale’, C = 0.001, decision_function_shape = ‘ovo’, tolerance = 0.0001, class weight = ‘balanced’, and jackknife = 0.25.

To ensure robustness, the entire workflow (data splitting, training, and parameter optimization) was repeated multiple times. The AUC (Area Under the ROC Curve) was computed for each run, and the reported values represent the highest AUCs obtained across repetitions.

Model performance was evaluated using ROC curves, with AUC results summarized in Figure A4 for the P4–P4′ region. Table A3 presents AUC values for all analyzed feature sets (P3–P4′, P4–P4′, and P15–P15′). Figure 10 shows pairwise AUC comparisons for the P3–P4′ region, which proved most informative for distinguishing cathepsin cleavage site specificities.

The combined data sets of two cathepsin substrates were also used for clustering, with the main goal being to obtain clusters of substrates from only one of the two cathepsins. The Ward method and the BLOSUM62 substitution matrix were used for classification as previously described [29]. The clustering variables were the specific positions from P3 to P4′. Clustering was performed for a maximum of 400 clusters. The clusters are listed in the Supplementary Data S1.

### 4.10. Docking

Protein–protein docking simulations were performed using the ClusPro 2.0 server [27]. Mutations were introduced using ChimeraX 1.10 [52], and both wild-type and mutant complexes were docked under identical conditions using ClusPro with and without specified interaction region. The resulting top-ranked docked complexes were further analyzed for interaction energy and hydrogen-bond networks at the interface using the MAIN software [26] and include van der Waals (Lennard–Jones potential) and electrostatic energy of interactions.

Cluster score from the ClusPro server [27] are presented in Table A5. Dockings with ID numbers 1357923 and 1358636 represent the mutated mCatB complex with DARPin 4m3, with and without specified interaction region, respectively (detailed coefficient weights are provided in Table A7). The “600Eelec” parameter set, known to provide reliable performance for enzyme–inhibitor complexes [27], was used. Docking results were validated using the wild-type complex of mCatB and DARPin 4m3, with (docking ID 1357926) and without (docking ID 1358637) specified interaction region (Table A6). Calculated interaction energies for our complex (molecules A/B and A2/B2), as well as for four docking models each of wild-type and mutated mCatB with DARPin 4m3, are shown in Table A8. Figure A6 displays the electrostatic model with code 002_00 (Table A7) for the complex of mutated mCatB (I65/Q66→S65/M66 substitution) and DARPin 4m3.

### 4.11. Data Availability

The CatB–DARPin 4m3 complex was deposited to the Protein Data Bank (PDB) and was assigned the entry 9S60.

Data sets of substrates of human CatK, V, B, L, S, F, and mCatB are available in the Supplementary Data S1.

### 4.12. Alignment and Comparison of CatB Sequences

The sequences of CatB from different animal species were compared using the Clustal Omega tool, v1.2.2) [53] and then colored in Jalview, v2.11.5.0 [54] according to the percent identity with the consensus sequence of nine animal species (rat, mouse, human, chicken, pig, cattle, sheep, orangutan, and macaque).

## Figures and Tables

**Table 1:** Kinetic and equilibrium constants for the interaction between mCatB and DARPin 4m3. Experimental values were obtained from the SPR data shown in Figure 2.

	k_a_ [1/Ms]	k_d_ [1/s]	K_D_ [nM]	χ^2^ [RU^2^]
mCatB pH 6	9.80 × 10^4^	6.44 × 10^−3^	65.7	0.021
mCatB pH 7	8.55 × 10^4^	9.25 × 10^−3^	108.3	0.004

**Table 2:** CETSA results showing temperatures at which mCatB reached 50% of its maximum band intensity on Western blots in the presence of DARPin E3_5, DARPin 4m3, DMSO, and CA074.

	DARPin E3_5	DMSO	DARPin 4m3	CA074
T_agg_ (50% of mCatB maximum band intensity)	52.1 °C	52.9 °C	62.9 °C	71.6 °C
95% confidence interval	51.6 °C–52.8 °C	52.3 °C–53.5 °C	62.39 °C–63.35 °C	70.7 °C–72.4 °C

**Figure A7:**
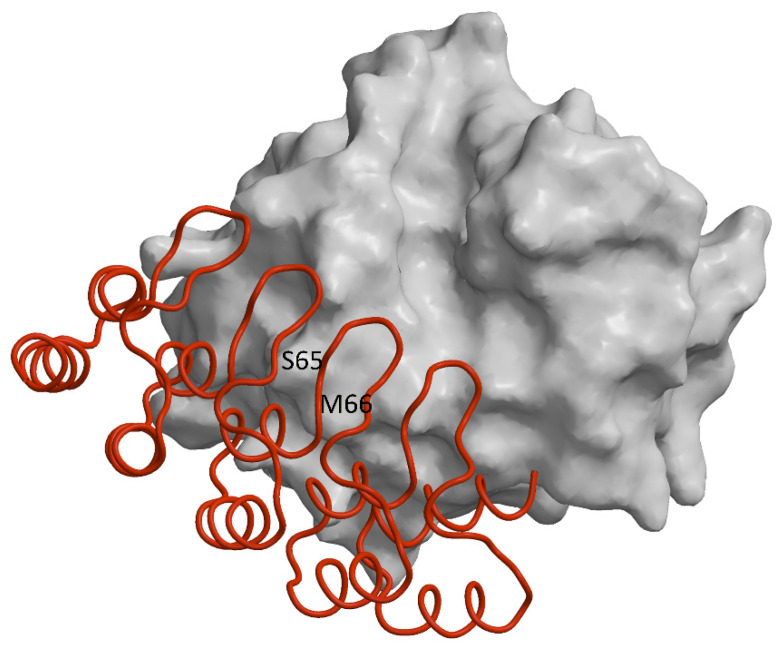
Docked complex of mutated mCatB (I65/Q66→S65/M66 substitution) and DARPin 4m3. The electrostatic model with code 002_00 (Table A7, red letters) is shown. Mutated mCatB is displayed as a white surface. DARPin 4m3 is shown as a red ribbon. The positions of the mutated residues in mCatB are labeled S65 and M66.

## Data Availability

The original contributions presented in this study are included in the article/Supplementary Materials. For further inquiries, please contact the corresponding author.

## Supplementary Materials

The following supporting information can be downloaded at: https://www.mdpi.com/article/10.3390/ijms262411910/s1.

## Abbreviations

The following abbreviations are used in this manuscript:

(m/h)Cat	(mouse/human) cathepsin
DARPin	designed ankyrin repeat proteins
nano-DSF	nano-differential scanning fluorimetry
SPR	surface plasmon resonance
CETSA	cellular thermal shift assay
AUC	area under curve
AMC	7-amino-4-methylcoumarin
ROC	receiver operating characteristic

## Appendix A

### Appendix A.2. Tables

**Table A1:** Structural data of the mCatB and DARPin 4m3 complex: (**a**) the data collection and refinement statistics and (**b**) the calculated RMSD between cathepsin B and DARPin molecules in the asymmetric unit and between human and mouse cathepsin B using superposition.

Data collection and refinement statistics.					
**PDB entry**					
**Data collection**					
Space group	P21 21 21				
Cell dimensions					
*a*, *b*, *c* (Å)	52.643, 109.921, 142.637				
	90.00, 90.00, 90.00				
Resolution (Å)	49.387–1.690 (1.67)				
*R*_means_	6.0 % (82.6 %)				
*I*/бꟾ	22.11 % (3.11 %)				
Completeness (%)	99.9 % (99.9 %)				
Redundancy	12.44 (12.54)				
**Refinement**					
Resolution (Å)	49.387–1.67				
No. reflections	96,907				
*R*_work_/*R*_free_	0.1931/0.2177				
No. atoms	14,655				
Protein	3741–3750				
Inhibitor	2262				
Water	2640				
*B*-factors	37.79				
Protein	36.77				
Inhibitor	35.08				
Water	49.34	Molecule 1	Molecule 2	RMSD (Å)	Sequence Identity (%)
R.m.s. deviations		Mouse cathepsin B	Human cathepsin B	0.70	83.1
Bond lengths (Å)	0.014	Mouse cathepsin B-molecule A	Mouse cathepsin B-molecule B	0.38	100
Bond angles (°)	1.527	Mouse cathepsin B-molecule A and DARPin B	Mouse cathepsin B-molecule B and DARPin B2	0.65	100
Data were collected from one crystal. Hydrogen atoms were excluded from the calculations. Values in parentheses are for the highest resolution shell.		DARPin B	DARPin B2	0.39	100
(**a**)		(**b**)			

**Table A2:** Summary of the results of pairwise structure alignment.

Protein 3D Structure 1	Protein 3D Structure 2	Alignment Length	RMSD	Sequence Identities (%)	Number of Different Residues	Number of Homologs
mCatB	hCatB	253	0.68	83.0	23	20
4m3	81	158	0.65	86.8	14	6
4m3	8h6	158	0.85	88.1	7	11
8h6	81	159	0.68	89.3	6	3

**Table A3:** Comparison of the combinations of pairs of data sets of the substrates of human cysteine cathepsins K, V, B, L, S, F and mouse cathepsin B by calculation of areas under the curve (AUC) of the ROC curve after using the Support Vector Machine (SVM) algorithm.

No	Cathepsin 1	Cathepsin 2	P3–P4′ (AUC)	P4–P4′ (AUC)	P15–P15′ (AUC)
1	mCatB	hCatB	0.7331	0.7922	0.9668
2	mCatB	hCatK	0.9386	0.9310	0.9722
3	mCatB	hCatV	0.9524	0.9616	0.9743
4	mCatB	hCatL	0.9542	0.9454	0.9754
5	mCatB	hCatS	0.9455	0.9524	0.9788
6	mCatB	hCatF	0.9653	0.9518	0.9745
7	hCatF	hCatB	0.9274	0.9360	0.9384
8	hCatK	hCatB	0.8759	0.9270	0.9042
9	hCatV	hCatB	0.9348	0.8291	0.9573
10	hCatL	hCatB	0.9069	0.8980	0.8884
11	hCatS	hCatB	0.9001	0.9192	0.9105
12	hCatF	hCatK	0.7404	0.7324	0.7744
13	hCatF	hCatL	0.6794	0.7406	0.7423
14	hCatK	hCatL	0.6735	0.7338	0.7325
15	hCatK	hCatV	0.7434	0.7759	0.9267
16	hCatK	hCatS	0.6667	0.7920	0.8163
17	hCatF	hCatS	0.7839	0.8788	0.8655
18	hCatF	hCatV	0.5470	0.6393	0.8667
19	hCatL	hCatV	0.6782	0.6557	0.6694
20	hCatS	hCatL	0.7487	0.7931	0.8082
21	hCatS	hCatV	0.7985	0.8387	0.8371

**Table A4:** Separate clusters (336, 339, 389, 396) with only mCatB substrates. The columns are UniProt code, sequential number of clusters among 400 clusters, positions from P3 to P4′, cleavage position in the protein and the name of protein.

UniProt	Cluster	P3	P2	P1	P1′	P2′	P3′	P4′	Cleavage (P1 Position)	Protein Name
Q3UWM4	336	K	K	G	M	A	T	A	916	Lysine-specific demethylase 7
Q80TJ7	336	K	K	G	L	A	T	A	1001	Histone lysine demethylase PHF8
Q9WTU0	336	K	K	G	M	A	T	A	1074	Lysine-specific demethylase PHF2
Q9R1Q8	339	Q	A	G	M	T	G	Y	188	Transgelin-3
Q9WVA4	339	Q	A	G	M	T	G	Y	188	Transgelin-2
P63325	389	K	K	A	E	A	G	A	140	40S ribosomal protein S10
Q61464	389	K	F	T	Q	A	G	A	395	Zinc finger protein 638
P39749	396	K	T	G	G	A	G	K	369	Flap endonuclease 1
P97386	396	K	R	G	T	A	G	C	102	DNA ligase 3

**Table A5:** Cluster scores (interaction energies between the two molecules) generated by the ClusPro server [27] are summarized for the docking runs performed with mutated mCatB (mut mCatB) and DARPin 4m3 (4m3) carrying the I65/Q66→S65/M66 substitution. Two docking jobs were used: one with (ID 1357923) and one without (ID 1358636) a specified interaction region. These results were validated against dockings of the wild-type mCatB–DARPin 4m3 complex performed under the same conditions, with (ID 1357926) and without (ID 1358637) the specified interaction region. For each docking, the coefficient weights for the interaction energy terms between the two proteins are listed. Here, Erep and Eattr represent the repulsive and attractive components of the van der Waals energy, respectively; Eelec denotes the electrostatic energy; and EDARS primarily reflects desolvation contributions. The parameter set “600Eelec” corresponds to the balanced weighting scheme, which has been shown to provide reliable performance for enzyme–inhibitor complexes [27].

		ID 1357923		ID 1357926		ID 1358636		ID 1358637	
		mut mCatB+ 4m3		mCatB+4m3 (PDB 9S60)		mut mCatB+4m3		mCatB+4m3 (PDB 9S60)	
		Specified Interaction Region		Specified Interaction Region					
	Coefficient Weights	E=0.40Erep+−0.40Eatt+600Eelec+1.00EDARS		E=0.40Erep+−0.10Eatt+600Eelec+0.00EDARS		E=0.40Erep+−0.40Eatt+600Eelec+1.00EDARS		E=0.40Erep+−0.40Eatt+600Eelec+1.00EDARS	
Representative	Cluster	Weighted score	Number of cluster members	Weighted score	Number of cluster members	Weighted score	Number of cluster members	Weighted score	Number of cluster members
Center	0	−1927.8	227	−407.7	249	−603.3	89	−603.5	87
Lowest Energy		−1991.6		−569.9		−633.8		−633.8	
Center	1	−1733.2	96	−321.1	107	−736.4	68	−715.5	85
Lowest Energy		−1797.4		−362.6		−840.9		−762.6	
Center	2	−1412.9	83	−318.4	101	−690.2	66	−736.4	66
Lowest Energy		−1628.9		−496.6		−690.2		−840.9	
Center	3	−1444.7	64	−334.2	85	−687.0	59	−690.3	65
Lowest Energy		−1570.3		−447.2		−687.0		−690.3	
Center	4	−1508.3	62	−359.4	77	−770.8	52	−686.8	56
Lowest Energy		−1887.9		−404.4		−770.8		−686.8	
Center	5	−1528.5	47	−365.4	62	−633.6	49	−762.3	51
Lowest Energy		−1621.3		−365.4		−762.3		−762.3	
Center	6	−1422.7	44	−312.8	43	−661.5	47	−770.8	49
Lowest Energy		−1560.4		−383.4		−758.2		−770.8	
Center	7	−1467.2	33	−333.9	39	−693.6	43	−693.6	41
Lowest Energy		−1650.4		−350.1		−708.4		−708.4	
Center	8	−1497.1	33	−308.6	34	−615.5	43	−615.8	39
Lowest Energy		−1653.3		−409.5		−615.5		−615.8	
Center	9	−1446.4	31	−313.0	33	−628.5	41	−628.0	38
Lowest Energy		−1624.5		−384.5		−628.5		−628.0	
Center	10	−1415.3	30	−315.3	32	−580.5	39	−581.0	37
Lowest Energy		−1665.5		−397.4		−635.7		−636.1	
Center	11	−1531.8	29	−311.3	29	−605.6	36	−605.6	37
Lowest Energy		−1645.1		−376.4		−697.9		−697.8	
Center	12	−1464.0	27	−336.5	25	−590.7	34	−590.7	33
Lowest Energy		−1673.4		−340.1		−692.7		−692.7	
Center	13	−1559.0	23	−323.1	21	−613.5	30	−613.8	33
Lowest Energy		−1646.4		−344.3		−675.2		−675.6	
Center	14	−1472.0	23	−330.9	18	−597.8	26	−576.4	26
Lowest Energy		−1625.1		−341.3		−680.2		−680.2	
Center	15	−1446.2	22	−330.0	14	−614.0	25	−614.3	24
Lowest Energy		−1446.2		−342.3		−630.8		−630.9	
Center	16	−1410.8	21	−321.3	13	−631.9	22	−592.0	20
Lowest Energy		−1513.6		−343.8		−647.9		−647.8	
Center	17	−1404.4	19	−327.5	12	−600.5	21	−600.8	20
Lowest Energy		−1678.2		−343.8		−636.0		−636.2	
Center	18	−1448.3	17			−571.1	18	−571.4	17
Lowest Energy		−1559.4				−679.8		−679.9	
Center	19	−1480.9	16			−642.9	15	−649.2	16
Lowest Energy		−1574.2				−642.9		−649.2	
Center	20	−1415.9	13			−602.4	14	−642.6	15
Lowest Energy		−1525.9				−668.9		−642.6	
Center	21	−1550.4	7			−596.3	14	−596.2	14
Lowest Energy		−1590.9				−634.0		−633.9	
Center	22	−1475.9	6			−579.3	13	−602.7	13
Lowest Energy		−1475.9				−607.5		−669.1	
Center	23	−1432.7	5			−655.1	13	−579.3	12
Lowest Energy		−1645.6				−655.1		−607.5	
Center	24	−1435.2	5			−626.3	13	−577.4	11
Lowest Energy		−1435.5				−626.3		−613.6	
Center	25					−577.4	12	−655.2	11
Lowest Energy						−613.5		−655.2	
Center	26					−598.9	10	−625.8	11
Lowest Energy						−708.5		−625.8	
Center	27					−581.8	9	−581.8	9
Lowest Energy						−604.5		−604.5	
Center	28					−568.6	5		
Lowest Energy						−647.8			

**Table A6:** Calculated hydrogen bonds between mCatB and DARPin 4m3 are shown for the four top-ranked docking complexes generated by the ClusPro server [27] (docking ID 1357926) and are compared with the hydrogen bonds identified in the experimentally determined complex (PDB 9S60). These docking models were used to validate the mutated mCatB–DARPin 4m3 complex (I65/Q66→S65/M66) presented in Table A7. ClusPro provides four sets of docking models based on different scoring schemes: balanced (000_00), electrostatic-favored (002_00), hydrophobic-favored (004_00), and van der Waals plus electrostatics (006_00). The docking complexes most similar to our complex (PDB code 9S60), based on hydrogen-bond patterns, are highlighted in cyan, magenta, red, and green. If a docking model lacked a hydrogen bond corresponding to one present in our complex, the respective cell was shaded with the appropriate solid color.

mouse cathepsin B				DARPin 4m3					000_00	006_00	002_00	004_00
Seq.1	Atom	Res.	Seq.2	Seq.3	Atom	Atom	Seq.4	**9S60**	**Bond length (Å)**	**Bond length (Å)**	**Bond length (Å)**	**Bond length (Å)**
896	O	CYS	62	4330	NE1	TRP	45					
906	O	CYS	63	3877	OG	SER	12					
906	O	CYS	63	4837	OH	TYR	79	2.77	2.6702	2.7861	2.6399	2.638
907	N	GLY	64	4330	NE1	TRP	45					
914	N	ILE	65	4816	OG	SER	78					
914	N	ILE	65	4837	OH	TYR	79	3.22	2.8232	3.4548	2.8894	3.0181
932	O	ILE	65	4219	NH2	ARG	37	2.98	2.8218	3.1575	3.2417	2.9154
933	N	GLN	66	4837	OH	TYR	79	2.91	2.871	3.2043	2.7597	2.7713
944	OE1	GLN	66	3772	NZ	LYS	5					
944	OE1	GLN	66	4216	NH1	ARG	37	3.34	2.6802	2.7271	2.6809	2.66
944	OE1	GLN	66	4701	OH	TYR	70					
944	OE1	GLN	66	4788	O	LEU	75					
944	OE1	GLN	66	4816	OG	SER	78					
945	NE2	GLN	66	3777	O	LYS	5					
945	NE2	GLN	66	3838	N	ASP	9					
945	NE2	GLN	66	3847	OD2	ASP	9					
945	NE2	GLN	66	4330	NE1	TRP	45	3.54				2.969
945	NE2	GLN	66	4837	OH	TYR	79					
949	O	GLN	66	4216	NH1	ARG	37	3	2.6756	2.6955	2.9473	2.8724
949	O	GLN	66	4219	NH2	ARG	37		3.3582	3.0714	3.1282	3.2709
966	O	GLY	68	4216	NH1	ARG	37					
966	O	GLY	68	4701	OH	TYR	70					
975	OD1	ASP	69	4216	NH1	ARG	37					
975	OD1	ASP	69	4219	NH2	ARG	37					
975	OD1	ASP	69	4676	O	TYR	68		3.3878			
975	OD1	ASP	69	4701	OH	TYR	70				2.9	2.7721
975	OD1	ASP	69	5112	OG	SER	99					3.2379
975	OD1	ASP	69	5116	N	LYS	100					
975	OD1	ASP	69	5132	NZ	LYS	100		2.7435	2.7872		
975	OD1	ASP	69	5175	ND2	ASN	103				3.061	3.0298
976	OD2	ASP	69	4183	ND1	HIS	35					
976	OD2	ASP	69	4216	NH1	ARG	37					
976	OD2	ASP	69	4650	NZ	LYS	67					
976	OD2	ASP	69	4655	O	LYS	67					
976	OD2	ASP	69	4673	OH	TYR	68					
976	OD2	ASP	69	4701	OH	TYR	70				3.0169	
976	OD2	ASP	69	5112	OG	SER	99				2.8871	2.8617
976	OD2	ASP	69	5116	N	LYS	100				3.4947	
976	OD2	ASP	69	5132	NZ	LYS	100		2.9037	2.8426		
976	OD2	ASP	69	5138	N	TYR	101				3.0628	3.1699
976	OD2	ASP	69	5175	ND2	ASN	103					
1005	OD1	ASN	72	5155	OH	TYR	101		2.7815	2.5237		
1005	OD1	ASN	72	5132	NZ	LYS	100					
1006	ND2	ASN	72	5155	OH	TYR	101					
1010	O	ASN	72	4650	NZ	LYS	67					
1010	O	ASN	72	5132	NZ	LYS	100					3.1431
1018	N	GLY	74	5155	OH	TYR	101					
1018	N	GLY	74	4673	OH	TYR	68					
1024	O	GLY	74	4183	ND1	HIS	35		2.6731	2.754		
1024	O	GLY	74	4673	OH	TYR	68	3.14		2.6048	2.6226	2.6693
1042	OH	TYR	75	4162	OD2	ASP	33	2.58	2.9256	2.8356	2.8501	2.8709
1042	OH	TYR	75	4192	O	HIS	35					
1042	OH	TYR	75	4330	NE1	TRP	45					
1042	OH	TYR	75	4630	OD1	ASP	66					
1067	OG	SER	77	4162	OD2	ASP	33				3.0181	
1060	N	SER	77	4189	NE2	HIS	35					3.447
1067	OG	SER	77	4189	NE2	HIS	35					2.9973
1077	O	GLY	78	4837	OH	TYR	79					
1119	OG	SER	81	3880	O	SER	12		3.427			
1119	OG	SER	81	4330	NE1	TRP	45	3.04		2.77		
1119	OG	SER	81	3847	OD2	ASP	9					
1119	OG	SER	81	3877	OG	SER	12	3.03			2.7744	2.7806
1122	O	SER	81	4330	NE1	TRP	45		2.8007		3.1864	
1122	O	SER	81	4837	OH	TYR	79					
1123	N	PHE	82	4837	OH	TYR	79					
1197	NZ	LYS	85	3846	OD1	ASP	9					
1197	NZ	LYS	85	3849	O	ASP	9					
1197	NZ	LYS	85	3877	OG	SER	12					
1197	NZ	LYS	85	3880	O	SER	12	2.71	2.8243		2.8216	2.7342
1197	NZ	LYS	85	3890	O	ALA	13	3.46	2.9464		2.8628	2.7085
1197	NZ	LYS	85	3950	OE1	GLU	18					
1197	NZ	LYS	85	3951	OE2	GLU	18					
1197	NZ	LYS	85	4342	O	TRP	45			2.7365		2.7946
1197	NZ	LYS	85	4353	O	SER	46		2.8315	3.0939	2.8378	2.959
1197	NZ	LYS	85	4840	O	TYR	79					
1197	NZ	LYS	85	4862	NE2	HIS	81			2.9283		
1197	NZ	LYS	85	5327	NE2	HIS	114					
1219	NZ	LYS	86	3859	O	ALA	10					
1219	NZ	LYS	86	3880	O	SER	12					
1219	NZ	LYS	86	3890	O	ALA	13					
1219	NZ	LYS	86	3897	O	GLY	14					
1219	NZ	LYS	86	3909	OE1	GLN	15					
1219	NZ	LYS	86	4342	O	TRP	45					
1219	NZ	LYS	86	4353	O	SER	46					
1219	NZ	LYS	86	4840	O	TYR	79		2.9024			
1219	NZ	LYS	86	5294	O	THR	111					
1219	NZ	LYS	86	5305	O	SER	112					
1219	NZ	LYS	86	5757	OD2	ASP	144					
1219	NZ	LYS	86	5768	OD1	ASN	145					
1219	NZ	LYS	86	5789	OD1	ASN	147					
1723	OE1	GLU	122	5132	NZ	LYS	100					2.61
1724	OE2	GLU	122	5132	NZ	LYS	100					2.778
1724	OE2	GLU	122	5155	OH	TYR	101				2.9329	2.8396
1742	OD1	ASP	124	5769	ND2	ASN	145					
1743	OD2	ASP	124	5769	ND2	ASN	145					
1743	OD2	ASP	124	5155	OH	TYR	101					
1759	O	THR	125	5769	ND2	ASN	145					
1746	N	THR	125	5155	OH	TYR	101					
1752	OG1	THR	125	5155	OH	TYR	101					
1787	NE	ARG	127	5768	OD1	ASN	145					
1787	NE	ARG	127	4819	O	SER	78					
1787	NE	ARG	127	5610	O	ALA	134					
1790	NH1	ARG	127	4840	O	TYR	79					
1790	NH1	ARG	127	5327	NE2	HIS	114					
1790	NH1	ARG	127	5756	OD1	ASP	144		3.4703	2.7657		
1790	NH1	ARG	127	5759	O	ASP	144	3.38	2.9402		2.8585	2.8483
1790	NH1	ARG	127	5768	OD1	ASN	145				2.7478	2.6823
1790	NH1	ARG	127	5773	O	ASN	145					
1790	NH1	ARG	127	5780	O	GLY	146					
1793	NH2	ARG	127	5756	OD1	ASP	144		2.8136			
1793	NH2	ARG	127	5757	OD2	ASP	144		2.8135			
1793	NH2	ARG	127	5768	OD1	ASN	145					
1793	NH2	ARG	127	5773	O	ASN	145					
1793	NH2	ARG	127	5780	O	GLY	146					
1793	NH2	ARG	127	5789	OD1	ASN	147					
1793	NH2	ARG	127	5756	OD1	ASP	144			2.7293		
1793	NH2	ARG	127	4819	O	SER	78					
1793	NH2	ARG	127	5305	O	SER	112					
1793	NH2	ARG	127	5327	NE2	HIS	114					
1793	NH2	ARG	127	5757	OD2	ASP	144				2.6142	
1793	NH2	ARG	127	5759	O	ASP	144	3.32			2.8846	
1793	NH2	ARG	127	5610	O	ALA	134					
1793	NH2	ARG	127	5617	O	GLY	135					
1793	NH2	ARG	127	5759	O	ASP	144					2.9702
1838	NZ	LYS	130	5756	OD1	ASP	144					
1838	NZ	LYS	130	5757	OD2	ASP	144					
1838	NZ	LYS	130	4353	O	SER	46					
1838	NZ	LYS	130	5759	O	ASP	144					
1838	NZ	LYS	130	5768	OD1	ASN	145					
1991	NZ	LYS	141	5759	O	ASP	144					
1991	NZ	LYS	141	5773	O	ASN	145					
1991	NZ	LYS	141	5780	O	GLY	146					
2136	O	SER	150	3772	NZ	LYS	5					
2158	N	SER	152	3846	OD1	ASP	9		2.8916	2.8338	2.9605	2.9852
2158	N	SER	152	3847	OD2	ASP	9	3.05	3.0655		2.9639	3.1333
2165	OG	SER	152	3846	OD1	ASP	9		2.8168	2.7938	2.9298	2.9162
2165	OG	SER	152	3847	OD2	ASP	9				3.0765	
2165	OG	SER	152	3877	OG	SER	12					
2165	OG	SER	152	3880	O	SER	12					
2165	OG	SER	152	4162	OD2	ASP	33					
2168	O	SER	152	3847	OD2	ASP	9			2.9045		
2168	O	SER	152	3772	NZ	LYS	5					3.4027
2184	O	VAL	153	3772	NZ	LYS	5	3.37		2.6812	2.9603	
2185	N	SER	154	3772	NZ	LYS	5					3.3823
2192	OG	SER	154	3794	NZ	LYS	6		2.688	2.561		
2192	OG	SER	154	3910	NE2	GLN	15					
2192	OG	SER	154	3772	NZ	LYS	5					2.8022
2204	OD1	ASN	155	3726	OD1	ASP	2					
2204	OD1	ASN	155	3794	NZ	LYS	6					
2205	ND2	ASN	155	3726	OD1	ASP	2					
2205	ND2	ASN	155	3727	OD2	ASP	2					
2205	ND2	ASN	155	3847	OD2	ASP	9					
2205	ND2	ASN	155	3727	OD2	ASP	2					
2205	ND2	ASN	155	3794	NZ	LYS	6					
2205	ND2	ASN	155	3847	OD2	ASP	9					
2209	O	ASN	155	3794	NZ	LYS	6					
3482	ND2	ASN	238	3726	OD1	ASP	2					
3482	ND2	ASN	238	3727	OD2	ASP	2					
3482	ND2	ASN	238	3772	NZ	LYS	5					
3564	OG	SER	244	3772	NZ	LYS	5					
3567	O	SER	244	3772	NZ	LYS	5		2.6728	3.0517	3.4348	
3567	O	SER	244	3846	OD1	ASP	9					
3567	O	SER	244	3847	OD2	ASP	9					
3579	OE1	GLU	245	3772	NZ	LYS	5		2.5268			
3579	OE1	GLU	245	4165	N	ALA	34		3.1376	2.7023	3.4453	
3579	OE1	GLU	245	4174	O	ALA	34				3.3716	
3579	OE1	GLU	245	4175	N	HIS	35			3.0971	3.1293	
3579	OE1	GLU	245	4183	ND1	HIS	35					
3579	OE1	GLU	245	4189	NE2	HIS	35				3.4091	
3579	OE1	GLU	245	4650	NZ	LYS	67				2.807	2.5528
3580	OE2	GLU	245	4165	N	ALA	34		2.8597			
3580	OE2	GLU	245	4175	N	HIS	35		2.762	3.3268		
3580	OE2	GLU	245	4183	ND1	HIS	35					2.85
3580	OE2	GLU	245	4189	NE2	HIS	35	2.9			3.3773	
3580	OE2	GLU	245	4650	NZ	LYS	67				2.8116	3.1788
3580	OE2	GLU	245	4673	OH	TYR	68					

**Table A7:** Hydrogen bonds were calculated for the mutated mCatB–DARPin 4m3 complex (I65/Q66→S65/M66) using the two highest-ranked docked models generated by the ClusPro server [27] (docking ID 1357923). ClusPro produces four scoring-based model sets; in this case, only two were used: Balanced (000_00), and Electrostatic-favored (002_00). Among these, the 002_00 model (highlighted in red) shows the greatest similarity in hydrogen-bond pattern to our complex (PDB code 9S60) and is therefore also illustrated in Figure A7.

**mutated mCatB**				**DARPin 4m3**				000_00	002_00
Seq.1	Atom	Res.	Seq.2	Seq.3	Atom	Atom	Seq.4	Bond length (Å)	Bond length (Å)
886	O	THR	61	4322	NE1	TRP	45		
906	O	CYS	63	4829	OH	TYR	79	2.5494	2.5218
906	O	CYS	63	3869	OG	SER	12		
914	N	SER	65	4829	OH	TYR	79	3.023	2.9128
914	N	SER	65	5167	ND2	ASN	103		
921	OG	SER	65	4280	O	HIS	41		
921	OG	SER	65	4281	N	ALA	42		
921	OG	SER	65	4761	O	HIS	74	2.8814	
921	OG	SER	65	4808	OG	SER	78	3.4602	2.8349
921	OG	SER	65	4829	OH	TYR	79		3.4503
921	OG	SER	65	4854	NE2	HIS	81		
921	OG	SER	65	5166	OD1	ASN	103		
921	OG	SER	65	5167	ND2	ASN	103		
924	O	SER	65	4211	NH2	ARG	37	3.335	2.7094
925	N	MET	66	4829	OH	TYR	79	2.6753	2.697
941	O	MET	66	4208	NH1	ARG	37	2.8147	2.788
941	O	MET	66	4211	NH2	ARG	37	3.2862	
941	O	MET	66	4829	OH	TYR	79		
952	N	GLY	68	4211	NH2	ARG	37		
958	O	GLY	68	4693	OH	TYR	70		
958	O	GLY	68	4208	NH1	ARG	37		
967	OD1	ASP	69	4208	NH1	ARG	37		
967	OD1	ASP	69	4211	NH2	ARG	37		
967	OD1	ASP	69	4693	OH	TYR	70	3.0486	
967	OD1	ASP	69	4808	OG	SER	78		
967	OD1	ASP	69	5104	OG	SER	99		
967	OD1	ASP	69	5167	ND2	ASN	103	3.098	
967	OD1	ASP	69	5124	NZ	LYS	100		2.8428
968	OD2	ASP	69	5124	NZ	LYS	100		3.1307
968	OD2	ASP	69	4208	NH1	ARG	37		
968	OD2	ASP	69	4211	NH2	ARG	37		
968	OD2	ASP	69	4693	OH	TYR	70	3.0127	
968	OD2	ASP	69	4808	OG	SER	78		
968	OD2	ASP	69	5104	OG	SER	99	2.8854	
968	OD2	ASP	69	5108	N	LYS	100		
968	OD2	ASP	69	5130	N	TYR	101	3.019	
997	OD1	ASN	72	5124	NZ	LYS	100		
997	OD1	ASN	72	5147	OH	TYR	101		2.8299
998	ND2	ASN	72	5147	OH	TYR	101		
997	OD1	ASN	72	5167	ND2	ASN	103		
1002	O	ASN	72	4693	OH	TYR	70		
1009	O	GLY	73	4665	OH	TYR	68		
1016	O	GLY	74	4665	OH	TYR	68	2.6996	2.5513
1016	O	GLY	74	4175	ND1	HIS	68		3.0273
1010	N	GLY	74	4665	OH	TYR	68		3.4597
1034	OH	TYR	75	4154	OD2	ASP	33	2.8301	2.8683
1034	OH	TYR	75	4153	OD1	ASP	33		
1059	OG	SER	77	4154	OD2	ASP	33	3.0075	
1059	OG	SER	77	4153	OD1	ASP	33		
1059	OG	SER	77	4175	ND1	HIS	35		
1069	O	GLY	78	4829	OH	TYR	79		
1111	OG	SER	81	3869	OG	SER	12	2.8087	2.7825
1114	O	SER	81	4322	NE1	TRP	45	2.9881	2.8261
1111	OG	SER	81	3838	OD1	ASP	9		
1111	OG	SER	81	3764	NZ	LYS	5		
1111	OG	SER	81	3838	OD1	ASP	9		
1111	OG	SER	81	3839	OD2	ASP	9		
1114	O	SER	81	3838	OD1	ASP	9		
1115	N	PHE	82	3838	OD1	ASP	9		
1189	NZ	LYS	85	3718	OD1	ASP	2		
1189	NZ	LYS	85	3719	OD2	ASP	2		
1189	NZ	LYS	85	3838	OD1	ASP	9		
1189	NZ	LYS	85	3839	OD2	ASP	9		
1189	NZ	LYS	85	3841	O	ASP	9		
1189	NZ	LYS	85	3872	O	SER	12	2.8225	2.7958
1189	NZ	LYS	85	3882	O	ALA	13	2.887	2.9848
1189	NZ	LYS	85	4334	O	TRP	45	3.2962	2.7322
1189	NZ	LYS	85	4345	O	SER	46	2.8047	2.7191
1189	NZ	LYS	85	4832	O	TYR	79		
1211	NZ	LYS	86	4832	O	TYR	79		2.9751
1211	NZ	LYS	86	5286	O	THR	111		
1211	NZ	LYS	86	5297	O	SER	112		
1211	NZ	LYS	86	5761	ND2	ASN	145		
1211	NZ	LYS	86	5781	OD1	ASN	147		
1211	NZ	LYS	86	3901	OE1	GLN	15		
1211	NZ	LYS	86	3942	OE1	GLU	18		
1211	NZ	LYS	86	3943	OE2	GLU	18		
1716	OE2	GLU	122	5147	OH	TYR	101	2.8632	
1715	OE1	GLU	122	5124	NZ	LYS	100		
1716	OE2	GLU	122	5124	NZ	LYS	100		
1715	OE1	GLU	122	5147	OH	TYR	101		
1735	OD2	ASP	124	5147	OH	TYR	101		
1734	OD1	ASP	124	5761	ND2	ASN	145		
1735	OD2	ASP	124	5761	ND2	ASN	145		
1735	OD2	ASP	124	5279	OG1	THR	111		
1735	OD2	ASP	124	4829	OH	TYR	79		
1738	N	THR	125	5147	OH	TYR	101		
1744	OG1	THR	125	5147	OH	TYR	101		
1738	N	THR	125	4829	OH	TYR	79		
1751	O	THR	125	4829	OH	TYR	79		
1779	NE	ARG	127	5602	O	ALA	134		
1779	NE	ARG	127	5765	O	ASN	145		
1782	NH1	ARG	127	5751	O	ASP	144	2.8501	2.8624
1782	NH1	ARG	127	5760	OD1	ASN	145	2.7165	2.7153
1782	NH1	ARG	127	5297	O	SER	112		
1782	NH1	ARG	127	4832	O	TYR	79		
1785	NH2	ARG	127	5748	OD1	ASP	144	2.6152	2.9704
1785	NH2	ARG	127	5749	OD2	ASP	144	2.6152	2.6974
1785	NH2	ARG	127	5751	O	ASP	144	2.8891	2.9265
1785	NH2	ARG	127	5602	O	ALA	134		
1785	NH2	ARG	127	5609	O	GLY	135		
1785	NH2	ARG	127	5286	O	THR	111		
1785	NH2	ARG	127	5765	O	ASN	145		
1785	NH2	ARG	127	5781	OD1	ASN	147		
1785	NH2	ARG	127	5319	NE2	HIS	114		
1830	NZ	LYS	130	5751	O	ASP	144		
1830	NZ	LYS	130	5760	OD1	ASN	145		
1830	NZ	LYS	130	3872	O	SER	12		
1830	NZ	LYS	130	4345	O	SER	46		
1983	NZ	LYS	141	5765	O	ASN	145		
1983	NZ	LYS	141	5772	O	GLY	146		
1983	NZ	LYS	141	3882	O	ALA	13		
1983	NZ	LYS	141	3889	O	GLY	14		
2125	OG	SER	150	3764	NZ	LYS	5		
2157	OG	SER	152	3764	NZ	LYS	5		
2150	N	SER	152	3838	OD1	ASP	9	2.9512	2.8781
2157	OG	SER	152	3838	OD1	ASP	9	2.9279	2.8744
2150	N	SER	152	3839	OD2	ASP	9	2.9574	2.8958
2157	OG	SER	152	3839	OD2	ASP	9	3.1087	3.1892
2157	OG	SER	152	3869	OG	SER	12		
2157	OG	SER	152	3872	O	SER	12		
2157	OG	SER	152	4154	OD2	ASP	33		
2176	O	VAL	153	3764	NZ	LYS	5	2.9539	
2184	OG	SER	154	3902	NE2	GLN	15		
2184	OG	SER	154	3786	NZ	LYS	6		2.6395
2197	ND2	ASN	155	3839	OD2	ASP	9		
2847	O	GLY	198	5124	NZ	LYS	100		
3559	O	SER	244	3764	NZ	LYS	5	3.426	
3556	OG	SER	244	3764	NZ	LYS	5		
3559	O	SER	244	3764	NZ	LYS	5		2.7417
3559	O	SER	244	3838	OD1	ASP	9		
3571	OE1	GLU	245	4157	N	ALA	34	3.4619	2.876
3572	OE2	GLU	245	4157	N	ALA	34		
3571	OE1	GLU	245	4166	O	ALA	34	3.3640	
3571	OE1	GLU	245	4167	N	HIS	35	3.0915	
3571	OE1	GLU	245	4181	NE2	HIS	35	3.4298	
3571	OE1	GLU	245	4642	NZ	LYS	67	2.8035	
3572	OE2	GLU	245	4181	NE2	HIS	35	3.3363	
3572	OE2	GLU	245	4642	NZ	LYS	67	2.7912	
3571	OE1	GLU	245	3764	NZ	LYS	5		2.7712
3572	OE2	GLU	245	3764	NZ	LYS	5		2.8691
3571	OE1	GLU	245	4642	NZ	LYS	67		
3572	OE2	GLU	245	4181	NE2	HIS	35		
3572	OE2	GLU	245	4642	NZ	LYS	67		
3572	OE2	GLU	245	4665	OH	TYR	68		

**Table A8:** Interaction energies were calculated for the mCatB–DARPin 4m3 complex (wild-type) and for docked models of the wild-type and two mutated complexes (I65/Q66→S65/M66) using the MAIN program [26]. Both van der Waals interactions (Lennard–Jones potential) and electrostatic energies were evaluated. Interaction energies are presented for two molecular pairs derived from our complex: mCatB (molecule A) with DARPin 4m3 (molecule B), and mCatB (molecule A2) with DARPin 4m3 (molecule B2). For comparison, four top-ranked ClusPro-docked models were analyzed for both the wild-type complex (docking ID 1357926) and the mutated complex (docking ID 1357923). ClusPro provides models generated under four scoring schemes: Balanced (000_00), Electrostatic-favored (002_00), Hydrophobic-favored (004_00), and van der Waals plus electrostatics (006_00).

Complex	Van Der Waals Contribution	Electrostatic Contribution	Total
mCatB and DARPin 4m3 (PDB code 9S60), molecules A (mCatB) and B (DARPin 4m3)	−85.6724	−339.6830	−425.3554
mCatB and DARPin 4m3 (PDB code 9S60), molecules A2 (mCatB) and B2 (DARPin 4m3)	−94.5953	−444.2739	−538.8692
mCatB and DARPin 4m3, docking model 000_00, ID 1357926	−28.4291	−719.8384	−748.2675
mCatB and DARPin 4m3, docking model 002_00, ID 1357926	−64.7532	−555.6167	−620.3699
mCatB and DARPin 4m3, docking model 004_00, ID 1357926	−41.1605	−529.7449	−570.9054
mCatB and DARPin 4m3, docking model 006_00, ID 1357926	−27.8825	−316.9363	−344.8188
Mutated mCatB and DARPin 4m3, docking model 000_00, ID 1357923	−65.0729	−351.8250	−416.8979
Mutated mCatB and DARPin 4m3, docking model 002_00, ID 1357923	−32.7603	−516.1930	−548.9533
Mutated mCatB and DARPin 4m3, docking model 004_00, ID 1357923	−32.6505	−426.5532	−459.2037
Mutated mCatB and DARPin 4m3, docking model 006_00, ID 1357923	−10.2918	−416.8580	−427.1498
